# Elimination of Cas9-dependent off-targeting of adenine base editor by using TALE to separately guide deaminase to target sites

**DOI:** 10.1038/s41421-022-00384-4

**Published:** 2022-03-23

**Authors:** Yang Liu, Jizeng Zhou, Ting Lan, Xiaoqing Zhou, Yang Yang, Chuan Li, Quanjun Zhang, Min Chen, Shu Wei, Shuwen Zheng, Lingyin Cheng, Yuling Zheng, Liangxue Lai, Qingjian Zou

**Affiliations:** 1 CAS Key Laboratory of Regenerative Biology, Guangdong-Hong Kong Stem Cell and Regenerative Medicine Research Centre, Joint Research Laboratory on Stem Cell and Regenerative Medicine, Guangzhou Institutes of Biomedicine and Health, Chinese Academy of Sciences, Guangzhou, China; 2 Guangdong Provincial Key Laboratory of Large Animal Models for Biomedicine, School of Biotechnology and Health Sciences, Wuyi University, Jiangmen, China; 3 School of Life Sciences, University of Science and Technology of China, Hefei, China; 4 School of Biomedical and Pharmaceutical Sciences, Guangdong University of Technology, Guangzhou, China; 5 University of Chinese Academy of Sciences, Beijing, China; 6 Bioland Laboratory (Guangzhou Regenerative Medicine and Health Guangdong Laboratory), Guangzhou, China; 7 Research Unit of Generation of Large Animal Disease Models, Chinese Academy of Medical Sciences (2019RU015), Guangzhou, China; 8 State Key Laboratory of Conservation and Utilization of Bio-Resources in Yunnan and Center for Life Science, School of Life Sciences, Yunnan University, Kunming, China

**Keywords:** DNA damage and repair, Bioinformatics

Dear Editor,

Adenine base editor (ABE) enables single-nucleotide conversions from A to G efficiently without double-stranded DNA breaks. ABE is a powerful tool for targeted base editing in cells and organisms^1,2^. However, deamination with ABE system at undesired RNA or DNA sites can cause off-target effect, which is the major safety concern for its application to the production of genetically modified animals as well as gene therapy^3–5^. Cas9-independent genome-wide off-target mutations are supposed to be caused by random deamination, which were not found in ABE. Rational engineering of deaminase domains with ABE7.10^F148A^ mutation has successfully resulted in the complete absence of RNA off-target effect^4,6^. Cas9-dependent off-target is caused by the tolerance of a mismatch between gRNA and targeting sequence. The high-fidelity Cas domain variants or truncated sgRNAs could reduce the frequency of Cas9-dependent off-targets events^7,8^ to some extent, but cannot completely eliminate Cas9-dependent off-targeting due to the mismatch tolerance of sgRNA in the genome.

A transcription activator-like effector (TALE) has been used to guide SpCas9 with attenuated DNA-binding affinity together with sgRNA to increase sequence targeting range and improve precision^9^. Theoretically, however, the fusion of SpCas9 with TALE can only reduce Cas9-dependent DNA off-target effects but is not able to eliminate it. Another study found when TALE or ZF fused with deaminase AID, a deaminase itself enabling rarely dsDNA editing, it could site-specifically convert C to T with very low efficiency (2.5%) in human cells^10^. Therefore, it is not applicable for practical gene-editing work.

We designed a novel base-editing system for efficient base editing on-target sites free of Cas9-dependent DNA off-targeting in human cells. The ABE7.10 is composed of a wild-type tRNA adenosine deaminase (TadA^wt^) and an evolved TadA^*^ heterodimer (TadA^wt*^) fused to nCas9, which is guided to target sites by sgRNA. In our new base-editing system, TadA^wt*^ catalytic domain from ABE7.10 is transferred to the TALE (Fig. 1a), resulting in a new base editor TadA^wt*^-TALE. TadA^wt*^ catalytic domain is guided to the target sites by TALE. TadA^wt*^-TALE alone has no base-editing activity on the target sites, due to the inability to unwind the double helix. When D10A nicked Cas9 (nCas9) is guided to the target site close to the TALE target to open the DNA duplex and nick the targeted strand, TadA^wt*^-TALE can convert A to G on the on-target single strand (Fig. 1b).

**Fig. 1:**
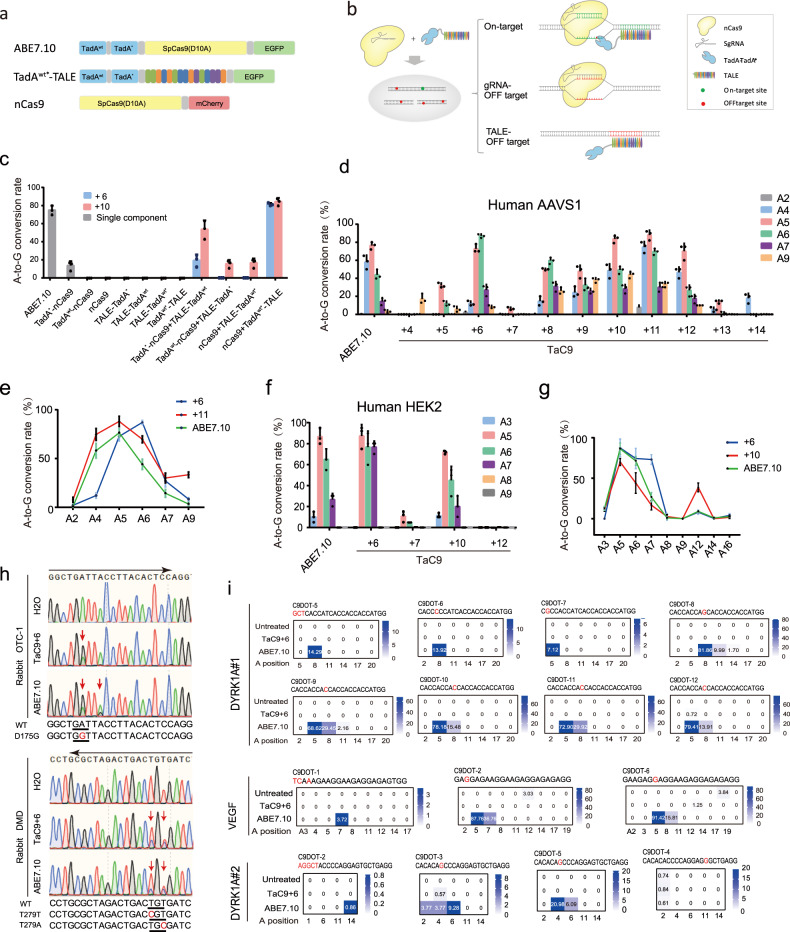
Engineering of TaC9-ABE with high on-target activity and without detectable Cas9-dependent off-target activity. **a** The main components of TaC9-ABE and ABE7.10 vectors. **b** Schematic diagram of the base-editing principle of TaC9-ABE at the target and off-target sites. Yellow shape indicates the nCas9 (S. pyogenes Cas9 with a D10A mutation). Colored clusters indicate TALE recognition sites. The blue pliers’ shape indicates the TadA wild-type and mutant dimer. Green dot and line indicate the on-target SgRNA sites. The red indicates the off-target sites. **c** The base-editing efficiency under different combinations of nCas9, adenosine deaminase, and TALE on human AAVS1 site. The gray bars represent the editing efficiency of a single component, and the uniform distance of the TALE component from the sgRNA site is 6 bp. The blue shows that the distance between TALE and sgRNA targets is 6 bp, and the pink represents 10 bp. **d**–**g** The base-editing efficiency of the TaC9-ABE system with a different target distance of TALEs and SgRNAs at AAVS1 (**d**) and HEK2 (**f**). Comparison of the base-editing efficiency of TaC9-ABE and ABE7.10 at AAVS1 site1 (**e**), HEK2 (**g**) in HEK293T cells (*n* = 3, means ± sem). **h** Representative Sanger sequencing chromatograms at endogenous OTC-1 and DMD loci from rabbit embryos. Red arrow: substituted nucleotide; black arrow: the gRNA direction; red letter: pathogenic mutation sites; WT: wild-type. The relevant codon identities at the target site are presented on the left of the DNA sequence. **i** The comparison of TaC9-ABE and ABE7.10 for the base-editing efficiency at Cas9-dependent off-target sites. Red letter indicates the off-target mismatch position. The blue shading indicates off-target editing frequencies. C9DOT: Cas9-dependent off-target. The average editing percentage derived from three independent experiments at the same site is listed.

With this system, when nCas9 is guided to a wrong site (mismatched off-target site) by sgRNA, base editing is not generated due to the absence of deaminase. Similarly, when deaminase is guided to a wrong site by TALE, the base editing will not happen due to the absence of ssDNA substrates. In this way, the Cas9-dependent off-target will be completely removed in our base-editing system.

To validate the target base-editing efficiency and effectiveness for overcoming off-target, three plasmids encoding TALE, nCas9, and sgRNA were constructed and co-transfected into HEK293T cells. EGFP and mCherry genes were fused to the TALE and nCas9 vectors, respectively. Cells positive for both EGFP and mCherry were selected by FACS, and analyzed by genomic PCR (Supplementary Fig. S1a).

To test the proper spatial position of adenosine deaminase in nCas9 and TALE, we fused the monomeric TadA^wt^ and TadA^*^ monomers, TadA^wt*^ heterodimer at the N-terminal of Cas9n, and the C- and N-terminal of TALE (Supplementary Fig. S1b). Human AAVS1 locus was chosen to test the editing efficiency with 6 and 10 bp spacers between nCas9 and TALE targets. As shown in Fig. 1c, ABE7.10 achieved at most ~75% A to G conversion. The separate expression of an individual component, such as TadA^wt^-nCas9, nCas9, TALE-TadA^*^, TALE-TadA^wt^, TALE-TadA^wt*^, and TadA^wt*^-TALE resulted in no detectable gene mutation on the target site, whereas TadA^*^-nCas9 had 18% A to G conversion. For two-component groups, TadA^wt^-nCas9 paired with TALE-TadA^*^ group showed 0% conversion when the spacer is 6 bp, and it showed 20% conversion when the spacer is 10 bp. In turn, TadA^*^-nCas9 paired with the TALE-TadA^wt^ group caused more effective editing (20 and 60%). The nCas9 paired with TALE-TadA^wt*^ group had limited base conversion efficiency (~20% at most). Fortunately, when nCas9 was combined with TadA^wt*^-TALE, the A to G conversion efficiency reached 80%, which was comparable with that of ABE7.10 (Fig. 1c). A new site of HEK2 was chosen to further prove the strong capability of nCas9 and TadA^wt*^-TALE group. When nCas9 was paired with TadA^wt*^-TALE (with 6 bp or 10 bp spacer), the base-editing efficiency was similar to or even higher than that of ABE7.10 (Supplementary Fig. S2). We named this base editor TaC9-ABE.

The distance of binding sites between nCas9 and TALE targets may affect editing efficiency. We tested the efficiency of TaC9-ABE with binding sites of different distances between nCas9 and TALE (11 bp; from 4 bp to 14 bp) on the AAVS1 gene to identify the optimal spacer lengths for base editing. Effective editing was achieved when spacer length ranged from 6 bp to 12 bp (Fig. 1d). TaC9-ABE with 6 bp spacer showed high editing efficiency at A5 and A6 sites, and the effective editing window (>20%) was narrowed from A5 to A7 (~3 nt). The larger spacers (9 bp to 11 bp) showed moderate base editing with a large editing window from A4 to A9 (~6 nt) (Fig. 1e, Supplementary Fig. S3a, b). We could hardly detect gene editing when the spacer length was 7 bp, which was confusing. We speculated that TALE failed to combine its target DNA due to the in-conformity of TALE design rule (upstream base should be T or C). To test this hypothesis, two genes (EMX1 and HEK2) in the human genome were used for TaC9-ABE-mediated base editing. Both targets showed similar rules as the AAVS1 site (Fig. 1f, Supplementary Figs. S3c–f, S4a). The spacer 7 bp, like spacer 6 bp, showed an effective editing window from A5 to A7 (~3 nt) (Supplementary Figs. S3f, S4a). It also showed that a larger spacer caused a larger editing window, and when the distance is too long, e.g., 12 bp at HEK2, gene-editing capability was not maintained (Fig. 1f, g). The results above suggested that to minimize the bystander editing, a reasonable spacer length between TALE and nCase9, such as 6 bp, should be chosen to narrow the editing window.

A previous study showed that adenosine deaminase F148A can achieve a more precise editing window and minimize RNA off-target effects^4^. However, when this genetic change was introduced into our TaC9-ABE, the editing window was truly narrowed to A5 and A6 compared with ABE7.10, while reduced efficiency was observed in positions other than A6 compared with its ordinary TaC9-ABE (Supplementary Fig. S5).

To prove the universality of TaC9-ABE among different cell lines and genes, we first targeted the EMX1 locus in Hela, U2-OS, and HEK293T cells. A6 and A7 were merely converted to G in the 44 bp target site (15 bp TALE target, 23 bp Cas9 target, and 6 bp spacer) (Supplementary Fig. S6a). No significant differences were found in base-editing efficiency among the three different cell lines (Supplementary Fig. S6b). Then, we tested the editing efficiency of TaC9-ABE among six more genes in the human genome and achieved the same results as those in the above experiments (Supplementary Fig. S6c).

To authenticate the editing capability of TaC9-ABE in vivo, we used this tool to perform A to G conversion in rabbit embryos. TaC9-ABE and ABE7.10 were designed to target rabbit OTC-1, OTC-2, and DMD loci. Compared with the control groups, blastocysts developed normally (Supplementary Fig. S7a) with a rate of 67%–77%. The rate of embryos with base editing was comparable with those in ABE7.10 groups (Supplementary Fig. S7b). Besides, to further prove the influence of the distance between nCas9 and TALE targets in vivo, we set two sgRNAs target sites (OCT-1 and OTC-2) with a distance of 6 and 10 bp respectively to the same TALE binding site on the OTC gene. As for 6 bp distance, the edit sites were limited on A6 at OTC-1 (resulted in codon GAT to GGT) by TaC9-ABE, while both A6 and A9 had been converted to G efficiently by ABE7.10 (codon GAT to GGT and TAC to TGC, Fig. 1h). As for 10 bp distance, the editing window of TaC9-ABE in OTC-2 was expanded from A2 to A12 (codon GAT to GGT, TAC to TGC, ACA to GCG) with the highest efficiency up to 98% on both A5 and A10, while the corresponding ABE7.10 only had editing activity on A5 (codon TAC to TGC, Supplementary Fig. S7c, d). At another target site DMD, the editing sites were limited on A5 and A7 by TaC9-ABE with a 6 bp distance (Fig. 1h and Supplementary Fig. S7d). The results above showed that TaC9-ABE also gave rise to high editing efficiency and a controllable editing window in embryo level and could be an effective tool for generation of animals with point mutations at specific sites.

To verify whether TaC9-ABE can overcome Cas9-dependent off-targeting, we edited seven sites in HEK293T cells. Total 8 target sites and their corresponding most likely off-target sites, 40 Cas9-dependent off-targets (C9DOTs) and 25 TALE-dependent off-targets (TaDOTs) were amplified from genomic DNA isolated from HEK293T cells treated with ABE7.10 or TaC9-ABE spaced 6 bp or 11 bp apart (Supplementary Data S1). High-throughput sequencing was performed to analyze on-target and off-target efficiency. As for on-targeting, TaC9-ABE showed the same or even higher base edit efficiency than ABE7.10 in all seven sites (Supplementary Fig. S8), whereas it did not result in any detectable genomic off-target modification in all 40-potential off-target sites (Fig. 1i, Supplementary Fig. S9, and Table S1). By contrast, half of the Cas9-dependent off-targets were observed with gene editing by 5 out of 7 gRNAs guiding ABE7.10, as follows: 1 out of 8 for EMX1; 3 out of 4 for VEGFA; 4 out of 5 for POU5F1; 10 of 11 for DYRK1A#1; and 2 of 6 for DYRK1A#2. To our surprise, ABE7.10 showed extremely high activity in half of the edited off-target sites (Fig. 1i, Supplementary Fig. S9 and Table S1). As for TALE-dependent off-targeting, TaC9-ABE did not result in any detectable genomic off-target modification in all 25 off-target sites of the 8 TALEs (Supplementary Tables S1, S2).

In summary, we described a new base-editing system, TaC9-ABE, which can completely eliminate Cas9-dependent off-targets without the cost of any targeting efficiency.

## Supplementary information

Supplementary information

Supplementary Table S2
