# Supplementary material for: Elimination of Cas9-dependent off-targeting of adenine base editor by using TALE to separately guide deaminase to target sites
Source: Cell Discov. 2022 Mar 23;8:28. doi: 10.1038/s41421-022-00384-4 (PMC8942999; doi:10.1038/s41421-022-00384-4)
Supplement: Supplementary file 1 — Supplementary information [file 41421_2022_384_MOESM1_ESM.docx]

Supplementary information

**Elimination of Cas9-dependent off-targeting of adenine base editor by using TALE to separately guide** **deaminase to the target site**

Yang Liu, Jizeng Zhou, Ting Lan, Xiaoqing Zhou, Yang Yang, Chuan Li, Quanjun Zhang, Min Chen, Shu Wei, Shuwen Zheng, Lingyin Cheng, Yuling Zheng, Qingjian Zou, Liangxue Lai

**Summary**

Supplementary information includes materials & methods, 9 figures, 2 tables (Table S2 is reserved as excel files) and the loci information.

**Materials and methods**

**Ethics statement.** The New Zealand White rabbits were purchased from the Laboratory Animal Center of the Southern Medical University (Guangzhou, China). All animal experiments were performed in accordance with the guidelines for care and use stipulated by Guangzhou Institute of Biomedicine and Health, Chinese Academy of Sciences (Animal Welfare Assurance #A5748-01), and effort was made to minimize animal suffering.

**Plasmid construction and in vitro transcription.** ABE7.10 and nCas9 were amplified from plasmid pCMV-ABE7.10 (Addgene, #102919) and inserted into pCDNA3.1, respectively. 2A-mCherry cassette was inserted following both genes to generate pcDNA3.1-ABE7.10-2aCherry and pcDNA3.1-nCas9-2aCherry. The TALE expressing plasmids (pE-SP6-TALE) were constructed by cloning the adenine deaminase fragments from pcDNA3.1-ABE7.10 into the N- or C- terminus of the modified plasmid pCAG-T7-TALEN (Sangamo)-FokI-ELD-Destination backbone (Addgene, #40132). 2A-EGFP cassette was inserted downstream of the TALE cassette. All TALE effectors were constructed by Golden Gate TALEN Assembly.

These vectors were constructed using a recombination kit (ClonExpress® MultiS, Vazyme). All sgRNAs used in this study were designed according to the N20-NGG rule. Two complementary sgRNA oligonucleotides were synthesized and annealed into double-stranded DNAs. Then, the annealed product was cloned into the U6-sgRNA cloning vector digested with BbsI to obtain a plasmid expressing sgRNA.

**mRNA and gRNA preparation.** pCDNA3.1 and pE vectors with T7 and SP6 promoters, respectively, were linearized with the restriction enzymes and purified with QiAquick PCR Purification Kit. Then, mRNAs were transcribed using HiScribe™ T7 ARCA mRNA kit (with tailing) (NEB) and mMessage mMachine SP6 Kit (Thermo Fisher/Ambion). RNA was recovered using the Rneasy MinElute Cleanup kit (Qiagen) and stored in aliquots at −80 °C. The annealed oligo sgRNAs were cloned into the pUC57-T7-sgRNA cloning vector (Addgene, #51306) and amplified in vitro. Then, gRNAs were generated using T7 High Yield RNA Synthesis Kit (NEB) and purified. All operations were performed according to the instruction manual.

**Cell culture and transfection.** HEK293T (ATCC CRL-3216), Hela (ATCC CCL-2) , and U2-OS (ATCC HTB-96) cells were cultured in DMEM (Gibco) supplemented with 10% fetal bovine serum (FBS, Gibco) and 1% (vol/vol) Penicillin-Streptomycin (Gibco) in a 37 °C humidified incubator with 5% CO_2_. Cells were passaged every 2–3 days when approximately 80%–90% confluence was reached. All experiments used cells before the 20th passage and tested for mycoplasma every 2 weeks. Here, 1.0 × 10^5^ cells were plated 24 h before transfection. The nCas9 expression plasmid (500 ng), TadA^wt*^-TALE plasmids (300 ng), and sgRNA plasmids (250 ng) were co-transfected into the experimental group cells, and ABE7.10 expression plasmid (500 ng) and sgRNA plasmids (250 ng) were co-transfected into control cells using Lipofectamine 8000 (Beyotime Biotechnology) according to the manufacturer’s protocol.

**Fluorescence-activated cell sorting.** Cells were washed with PBS and trypsinized with 0.05% trypsin-EDTA after 48 or 72 h post-transfection. They were resuspended in supplemented PBS at 1×10^6^ cells per milliliter and filtered through a 40-μm cell strainer for sorting. All EGFP- and mCherry-positive cells were obtained by fluorescence-activated cell sorting (FACS) (MoFlo Astrios, Beckman).

**Rabbit embryo injection.** The procedures involved in the microinjection of pronuclear stage embryos were performed in detail in published protocols^1^. The experimental group with nCas9-2a-cherry mRNA (150 ng/ul), TadA^wt*^-TALE-2a-GFP mRNA (100 ng/ul), and sgRNA (50 ng/ul) or the control group with ABE7.10 mRNA (150 ng/ul) and sgRNA (50 ng/ul) were co-injected into the cytoplasm of pronuclear-stage zygotes. The embryos were transferred to Earle's balanced salt solution medium and cultured at 38.5°C in 5% CO_2_.

**DNA extraction and targeted amplicon sequencing.** Injected embryos were collected at the blastocyst stage and lysed in 10 μL of lysis buffer (0.45% NP-40 plus 0.6% proteinase K) at 56 °C for 60 min and then at 95 °C for 10 min and then subjected to Sanger sequencing. Flow-sorted cells are identified using the same method. The target sequence was amplified from the genome of the transfected cells by PCR (2×PhantaMax Master mix, Vazyme) with specific primers. The products were subjected to Sanger and deep sequencing.

**Off-target analysis.** The potential POTs of each sgRNA were predicted in human genome to analyze site-specific edits by using an online design tool (http://www.rgenome.net/cas-offinder/)^1^. All POTs were amplified by PCR and then deep-sequenced to confirm off-target effects. All sites and their primers are listed in the supplementary information.

**Targeted deep sequencing.** Targeted sites were amplified from the extracted DNA with the corresponding site-specific primers using Q5 High-Fidelity DNA Polymerase (Takara). In this experiment, we used two deep sequencing platforms for off-target analysis, as follows. (i) For Hi-TOM platform, the identification primer design (18–2 nt) was carried out according to the conventional PCR primer design principles. The detection site must be within the range of 10–100 bp in the forward or reverse primer, and the bridging sequence 5-ggagtgagtacggtgtgc-3 should be added to the front of the forward primer. At the same time, the bridging sequence 5'-gagttggatgctggatgg-3' was added to the front end of the reverse primer. Each PCR was performed in 30 μL volume comprising 2 μL of the template according to the manufacturer's protocol. The paired-end (PE) reads with 150 bp sequencing of PCR amplicons were performed using an Illumina HiSeq platform (Illumina, USA). (ii) Deep sequencing libraries were constructed following the manufacturer’s protocol (TruePrepTM Index Kit V4 for Illumina, Vazyme). Each DNA sample was amplified using i5 and i7 primers and prepared by two PCR rounds. Both primers have a sequence that can anneal to the end of the first round of primers for bridge PCR, and the i7 primers both have a six-base index for multiplexing. All amplified products were electrophoresed in a 1% agarose gel with 1×TAE buffer. The target band was cut and recovered using HiPure Gel Pure DNA Mini Kit (Magen). DNA sample concentration was determined by Ipure Qubit dsDNA HS Assay Kit (IGE Biotech). Then, DNA samples at equal amounts of 50 ng were mixed to generate an Illumina sequencing library. The library was sent to Annoroad Gene Technology Corporation (Beijing) for deep amplicon sequencing using the Illumina HiSeqX platform and a paired-end read of 150 bp was generated. The original targeting sequence in the reads was analyzed to identify A-to-G. The ratio is calculated by comparing a single reading with the entire reads.

**Statistics and data reporting.** The average editing frequencies of Sanger sequencing in the editing window were analyzed on the online tool EditR 1.0.10^2^. Amplicon deep sequencing data were analyzed with CRISPResso2 v.2.0.30^3^. The GraphPad Prism software (version 8) was used for data analysis. The data are presented as the mean values± s.e.m. All tests conducted were two-tailed Student’s t-test, and the difference was considered significant at P < 0.05.

1 Bae, S., Park, J. & Kim, J. S. Cas-OFFinder: a fast and versatile algorithm that searches for potential off-target sites of Cas9 RNA-guided endonucleases. *Bioinformatics* **30**, 1473-1475 (2014).

2 Kluesner, M. G. *et al.* EditR: A Method to Quantify Base Editing from Sanger Sequencing. *CRISPR J* **1**, 239-250 (2018).

3 Clement, K. *et al.* CRISPResso2 provides accurate and rapid genome editing sequence analysis. *Nature biotechnology* **37**, 224-226 (2019).

**Supplementary figures**

**
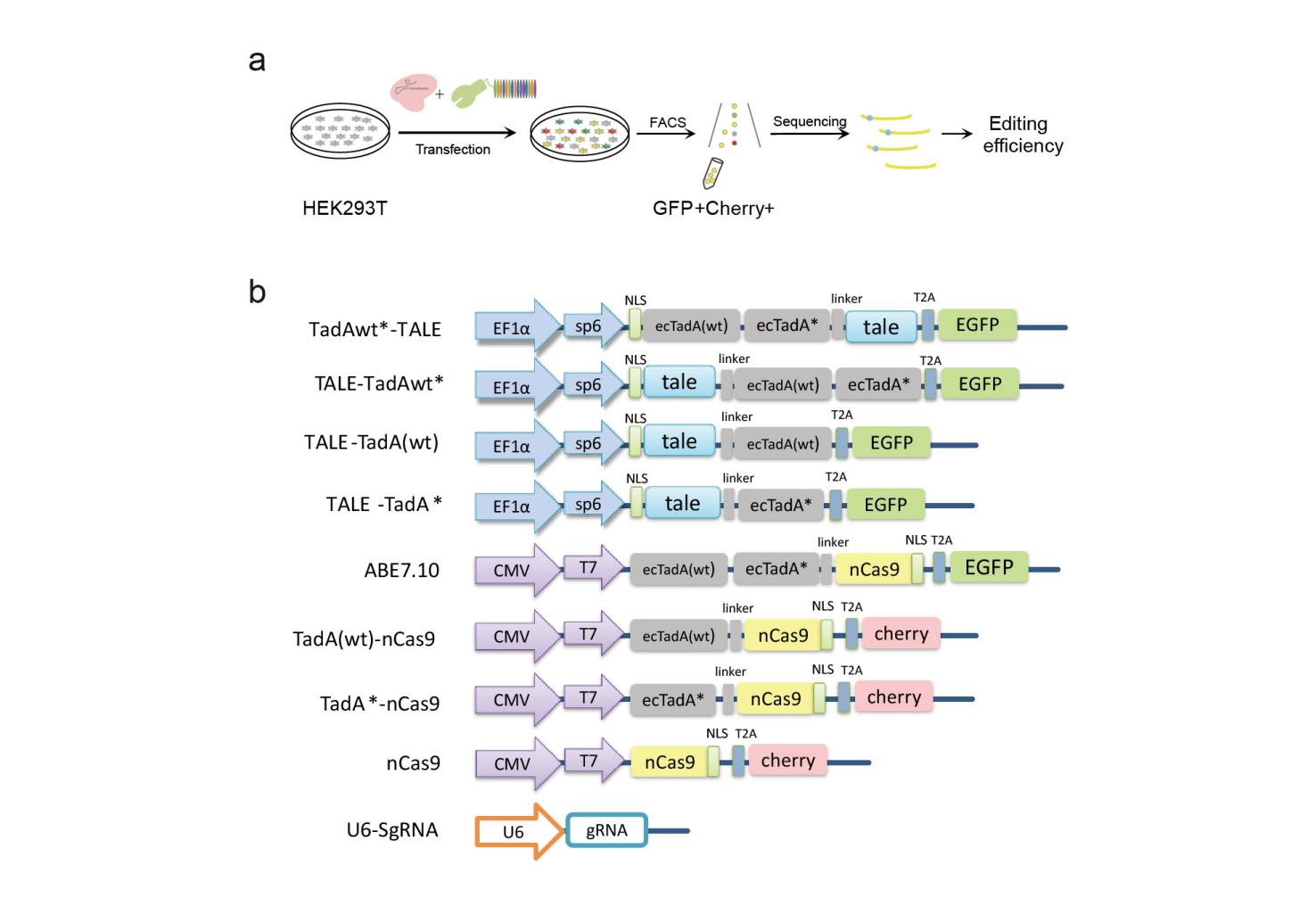
**

**Supplementary Figure S1.** **Schematic diagram of the experimental procedure and the structure of vectors used in this study.** (**a**) An overview of experimental testing of TaC9-ABE for target DNA sites. Cells carrying both mCherry and EGFP are indicated in yellow. The blue dots indicate sites of potential editing on DNA (yellow lines). (**b**) The structure of vectors used for the optimization of the TaC9-ABE system.

**
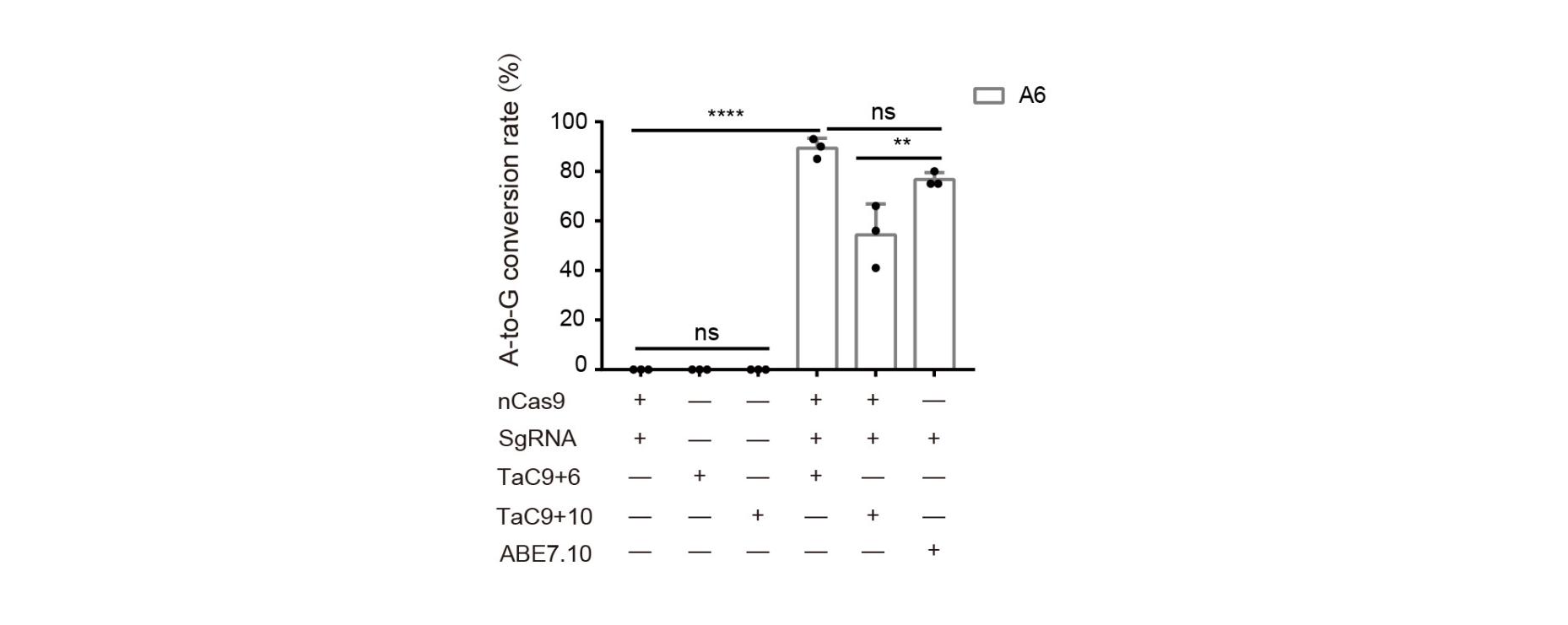
**

**Supplementary Figure S2.** **Base editing rate of the A6 of HEK2 site on various combinations of sgRNA, TaC9, and ABE7.10.** Data presented as mean ± s.e.m., **, p<0.01, ****, p<0.0001; ns, no significance, n = 3.

**
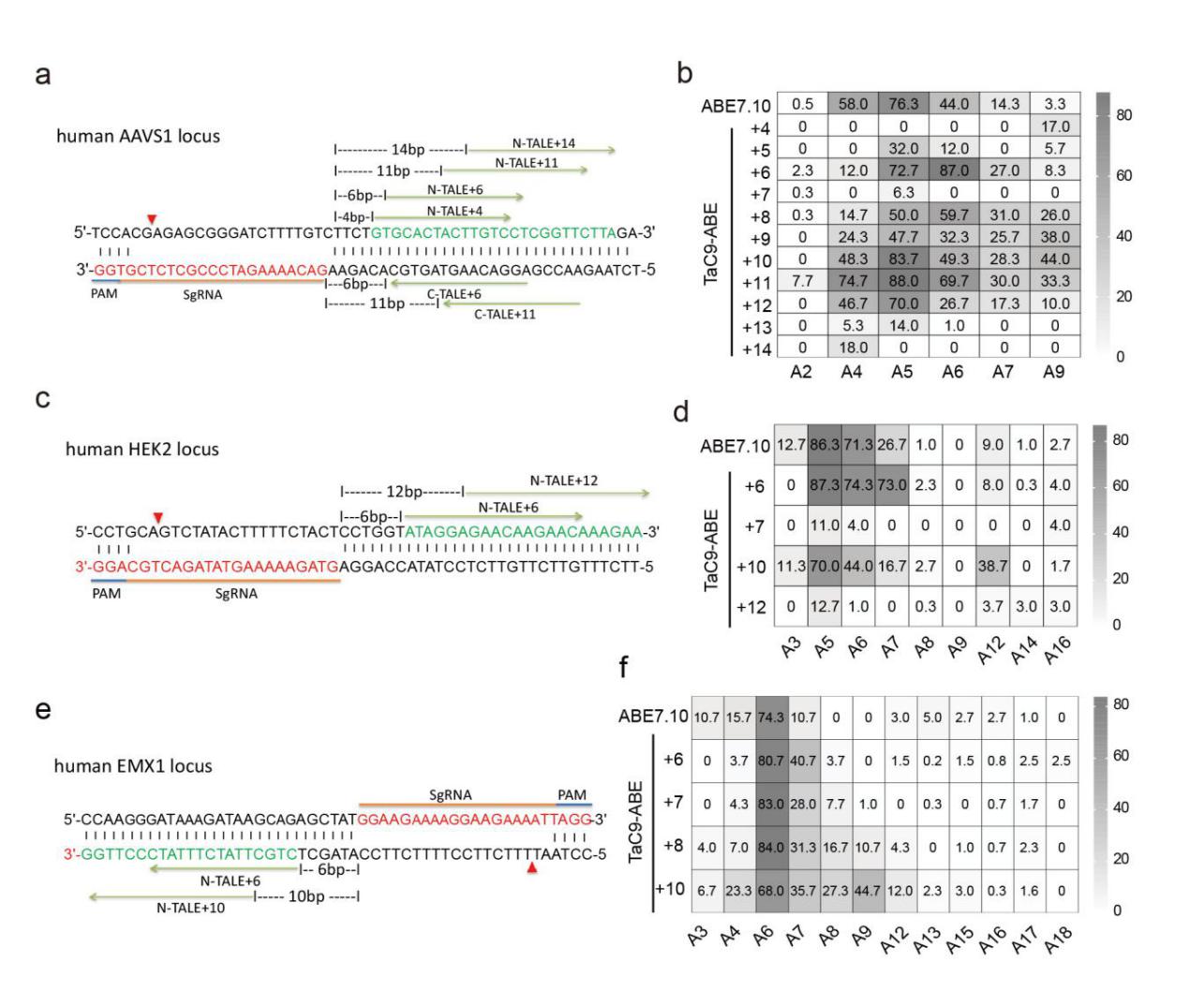
**

**Supplementary Figure S3.** **The relationship of base editing efficiency and the distance of targets between the two components of TaC9-ABE in HEK293T cells.** TALE targets (green) and SgRNA targets (red) at human AAVS1 (**a**), HEK2 (**c**), and EMX1 loci (**e**). N-TALE+6 means that TadA^wt*^ is at the N terminal of TALE, and the distance is +6 bp, whereas C-TALE means that TadA^wt*^ is at the C terminal. Single-strand break (red arrowhead). The PAM is shown in blue lines. The editing efficiencies of TaC9-ABE and ABE7.10 were examined at human AAVS1(**b**), HEK2 (**d**), and EMX1 loci (**f**). The average editing percentage derived from three independent experiments at the same site is listed.

**
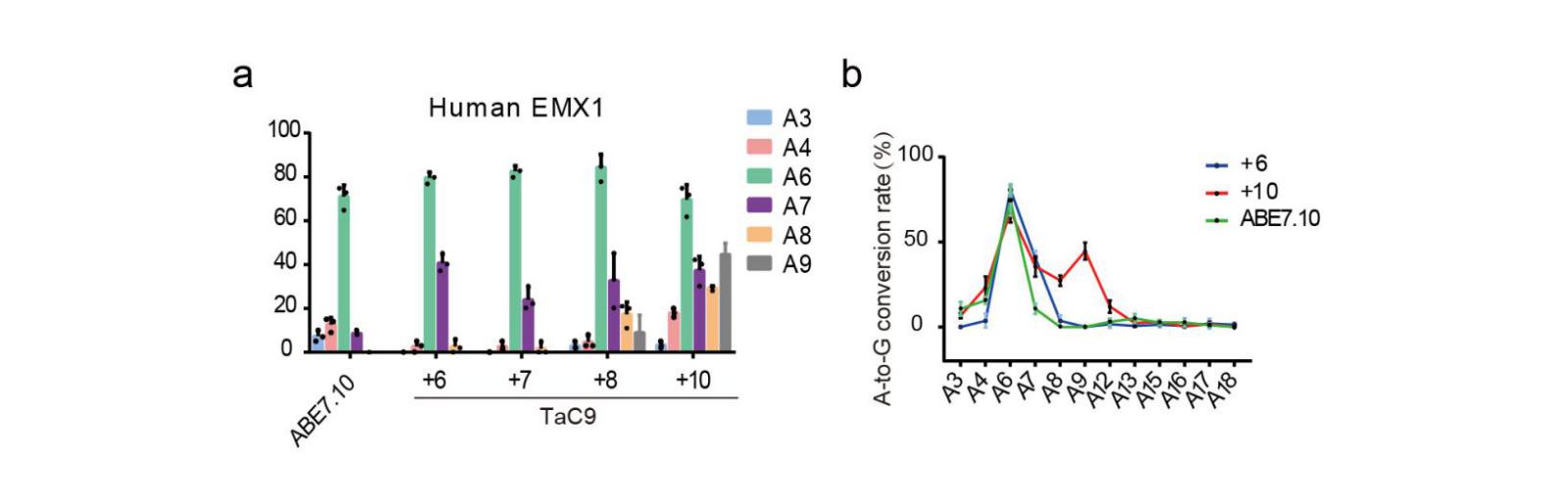
 Supplementary Figure S4.** **Base editing on EMX1 site of TaC9-ABE system in HEK293T cells.** (**a**)The base-editing efficiency of the TaC9-ABE system with a different target distance of TALEs and SgRNAs at EMX1 loci. (**b**)Comparison of the base editing efficiency of TaC9-ABE and ABE7.10 at EMX1 loci in HEK293T cells (n= 3, mean ± s.e.m.).

**
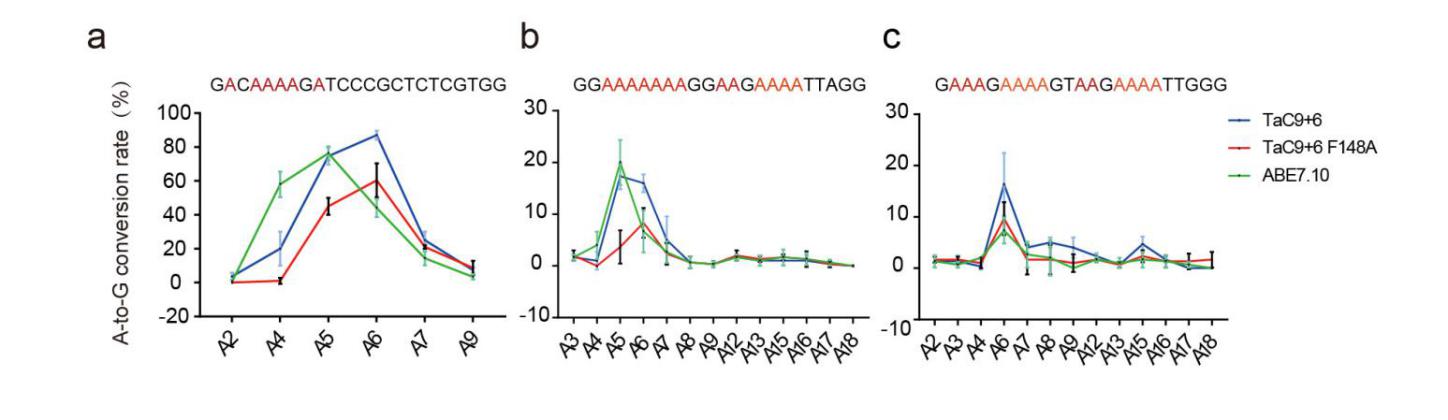
**

**Supplementary Figure S5.** **DNA on-target activities of TaC9-ABE F148A variants.**

The editing efficiency of TaC9-ABE, TaC9-ABE F148A, and ABE7.10 at AAVS1 (**a**), HEK3 (**b**), and HEK4 loci (**c**). Values and error bars reflect the mean ± s.e.m. and standard deviation of three independent biological replicates performed on different days.

**
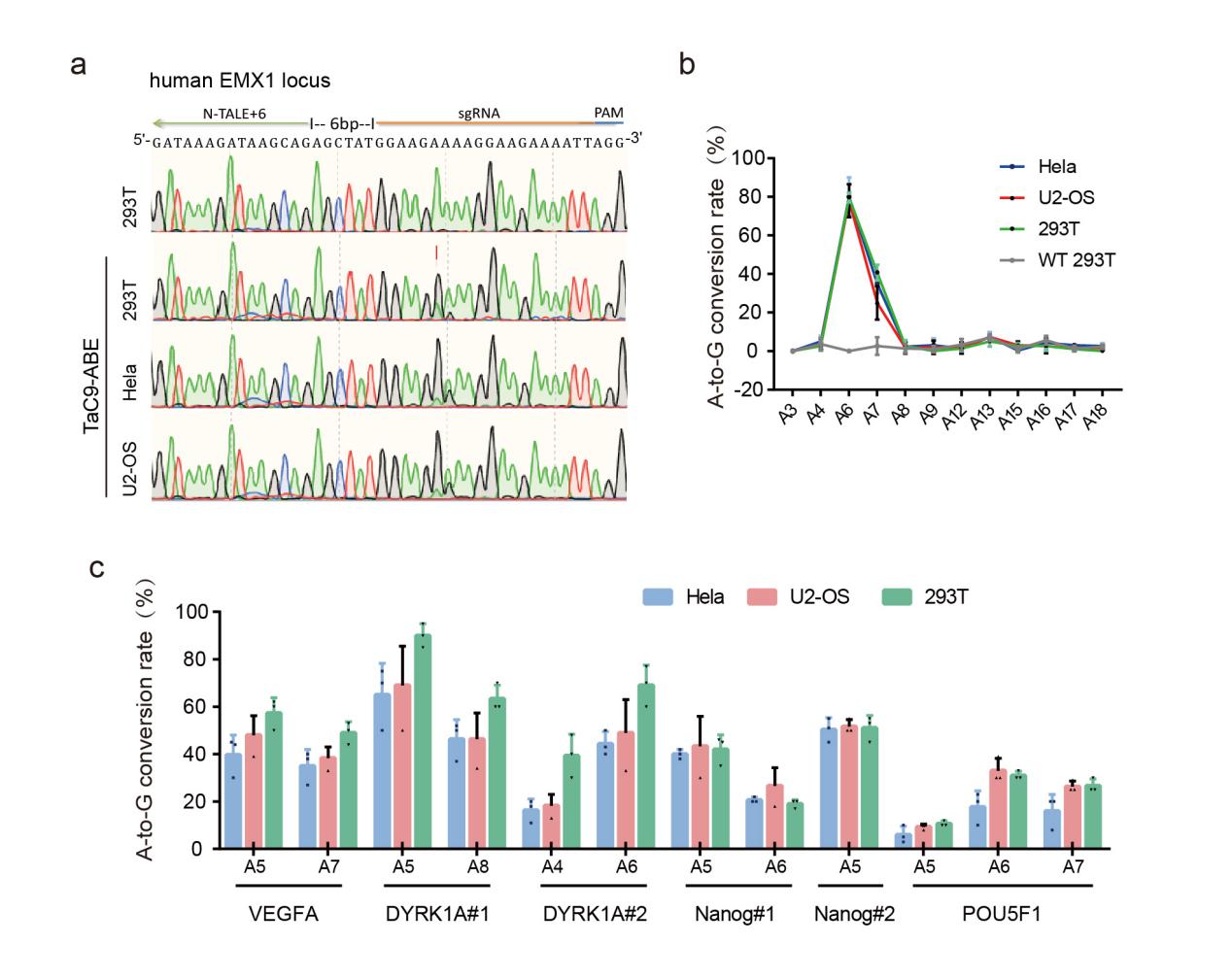
**

**Supplementary Figure S6.** **Base editing efficiencies of TaC9-ABE in HEK293T, Hela, and U2-OS cells.** (**a**) Sanger sequencing chromatograms from WT and three kinds of human cell lines. The red arrows indicate the A to G substituted nucleotides at human EMX1 locus. (**b**) Comparison of TaC9-ABE editing efficiency at three kinds of human cell lines. (**c**) Summary of the on-target editing efficiency of TaC9-ABE in HEK293T, Hela, and U2-OS cells at the six loci. Values and error bars reflect the mean± s.e.m and standard deviation of at least three biological experiments done on different days.

**
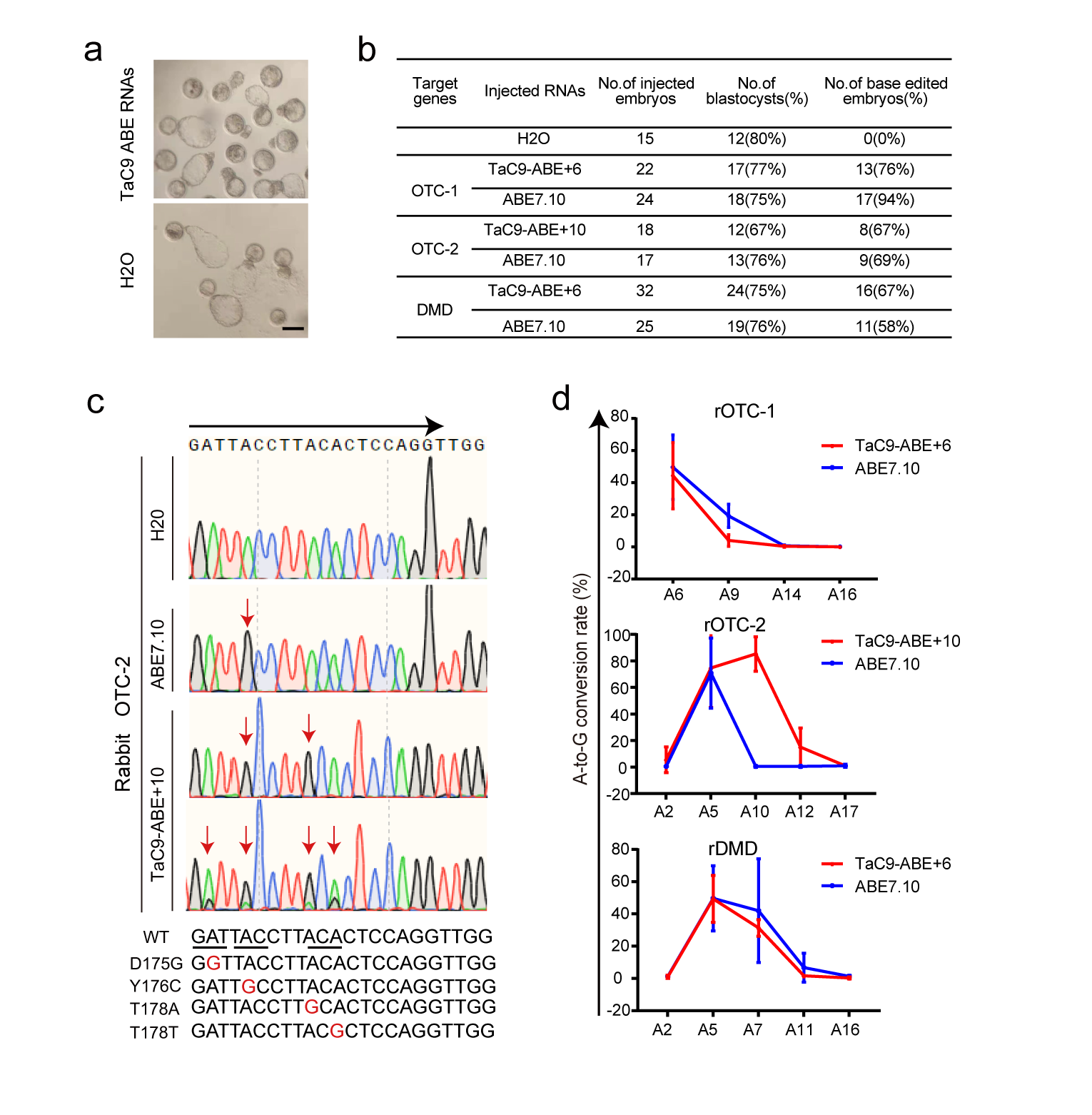
**

**Supplementary Figure S7.** **TaC9-ABE mediates efficient adenine editing in rabbit embryos.** (**a**) The edited E4.5 rabbit embryos after microinjected with TaC9-ABE RNA or H2O. Scale bar, 200 μm. (**b**) Summary of embryo development and editing rate using TaC9-ABE and ABE7.10 system. (**c**) Representative Sanger sequencing chromatograms at endogenous OTC-2 locus of rabbit embryos. Red arrow: overlapping peak sites; black arrow: the gRNA direction; red letter: pathogenic mutation sites; WT: wild-type. The relevant codon identities at the target sites are presented on the left of the DNA sequence. **(d) C**omparison of base editing efficiency of TaC9-ABE and ABE7.10 systems at rabbit OTC-1, OTC-2, and DMD loci. Data are presented as mean ± s.e.m.

**
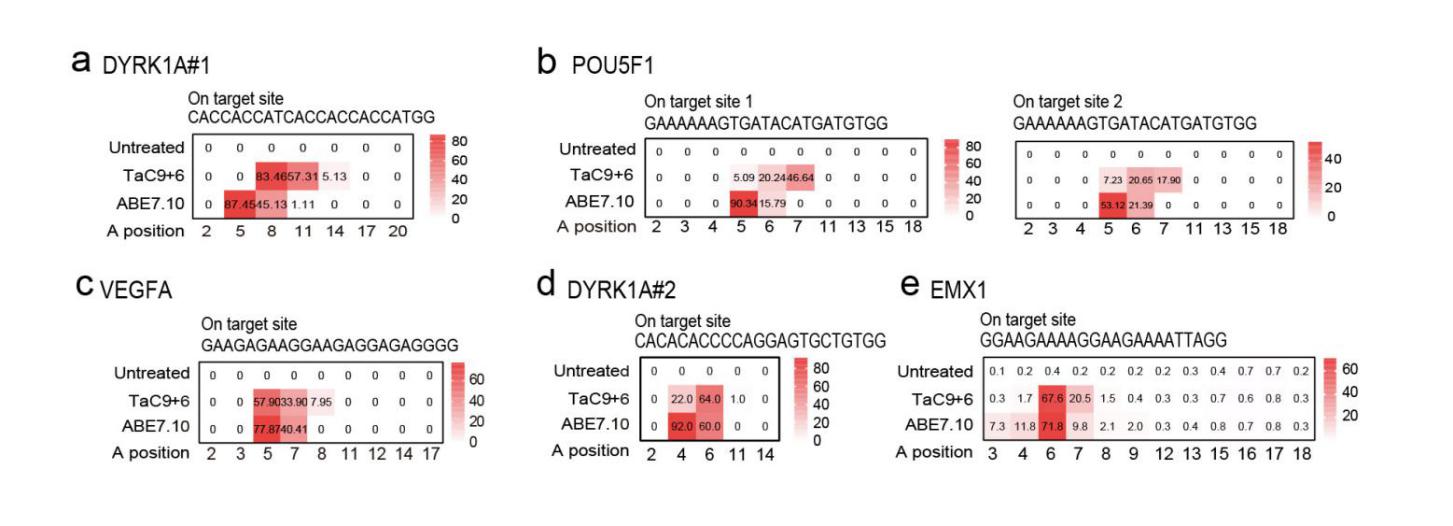
**

**Supplementary Figure S8. The comparison of TaC9-ABE and ABE7.10 for the base editing efficiency at Cas9-dependent on-target sites.** Red shading in the heat map indicates on-target editing frequencies.

**
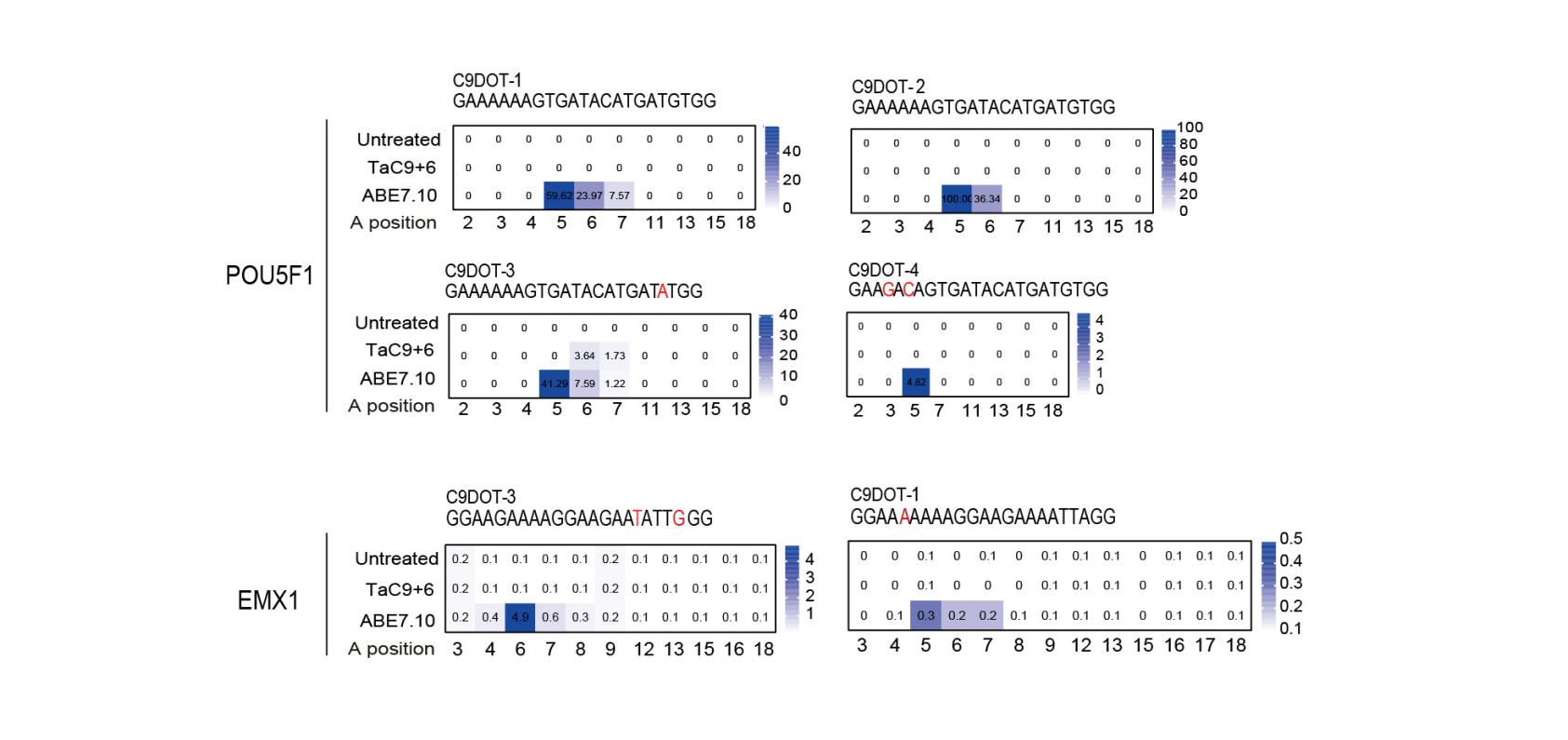
**

**Supplementary Figure S9.** **The comparison of TaC9-ABE and ABE7.10 for the base editing efficiency at Cas9-dependent off-target sites.** Red letter indicates the off-target mismatch position. The blue shading indicates off-target editing frequencies. C9DOT: Cas9-dependent off-target. The average editing percentage derived from three independent experiments at the same site is listed.

**Supplementary Table S1. Summary of on-targeting and off-targeting efficiency of TaC9-ABE**

|  |  | On targeting | | Cas9-dependent off-target editing | | | | TALE-dependent off-target editing | |
| --- | --- | --- | --- | --- | --- | --- | --- | --- | --- |
| Target genes | Base editing tools | ON target sites | Base editing | Identified C9DOTs | Total base editing C9DOTs | Base editing with high efficiency (>40%) | Base editing with moderate efficiency (<40%) | Identified TaDOTs | Total base editing |
| EMX1 | TaC9-ABE | 1 | 1 | 8 | 0 | 0 | 0 | 11 | 0 |
|  | ABE7.10 | 1 | 1 | 8 | 1 | 0 | 1 | / | / |
| VEGFA | TaC9-ABE | 1 | 1 | 4 | 0 | 0 | 0 | 0 | 0 |
|  | ABE7.10 | 1 | 1 | 4 | 3 | 2 | 1 | / | / |
| POU5F1 | TaC9-ABE | 2 | 2 | 5 | 0 | 0 | 0 | 1 | 0 |
|  | ABE7.10 | 2 | 2 | 5 | 4 | 3 | 1 | / | / |
| DYRK1A#1 | TaC9-ABE | 1 | 1 | 11 | 0 | 0 | 0 | 2 | 0 |
|  | ABE7.10 | 1 | 1 | 11 | 10 | 5 | 5 | / | / |
| DYRK1A#2 | TaC9-ABE | 1 | 1 | 5 | 0 | 0 | 0 | 2 | 0 |
|  | ABE7.10 | 1 | 1 | 5 | 2 | 0 | 2 | / | / |
| AAVS1 | TaC9-ABE | 1 | 1 | 4 | 0 | 0 | 0 | 6 | 0 |
|  | ABE7.10 | 1 | 1 | 4 | 0 | 0 | 0 | / | / |
| HEK2 | TaC9-ABE | 1 | 1 | 3 | 0 | 0 | 0 | 3 | 0 |
|  | ABE7.10 | 1 | 1 | 3 | 0 | 0 | 0 | / | / |
| Total | TaC9-ABE | 8 | 8 | 40 | 0 | 0 | 0 | 25 | 0 |
|  | ABE7.10 | 8 | 8 | 40 | 20 | 10 | 10 | / | / |

**The loci information of on-target and off-target sites**

NNNNNNNN: TALE target sites

NNNNNNNN: Cas9 target sites

**NNNNNNNN: mismatch sites**

NNNNNNNN: gene exon regions

1. Emx1

| TaC9 target locus | On target sites | gRNA off target sites | Tale off target sites |
| --- | --- | --- | --- |
| Emx1 | 1 | 8 | 11 |

on target sequence：GATAAAGATAAGCAGAGCTATggaagaaaaggaagaaaattagg

>chromosome:GRCh38:2:72914517:72915134:1

72914517 TGTCCAGTGGTGAAGAGGGGAAGTATTTGTGTATACGATCAGTTGTGGGGGATGGTGGTA 72914576

72914577 GAGGGAGCAGTATGAGAGTCTGGGAGGAACAGGGCAGGGGAGATGGCACAGGAGAAGATT 72914636

72914637 GGGGTGGGGGTGGGGGCACTGATACTGAATCCAGTACACAGAGGCAGGCTTAGAGACCCC 72914696

72914697 CTGAGGGTGACAAATTCTTCTCTTAACTTGCTGCAGAGGAAACGACAAGAGAATAGGTCC 72914756

72914757 TAAGAGGAGTGAAAATAGAGGAAACTGGCCAAGGGATAAAGATAAGCAGAGCTAT**GGAAG** 72914816

72914817 **AAAAGGAAGAAAATTAGG**GAATTACTGGAGAGGAAGCTGACAGAAAGGGGCGCTGGGAGA 72914876

72914877 GGAAAAGGTGAGGGAGGACGAAAACGGAACTCCTATCACCCAGCGCATTCCAGCACCCCC 72914936

72914937 TCCCCCTCCCCCGGGAAGTCTGCGACTGCATCCTCACAGGGATGGAATGGGAGTGGAAGC 72914996

72914997 TAGGCCAGGCTGGGACCCCGGGGCTGTCCCAGCCCAACCAAGACGGTGACCAGACTCAAG 72915056

72915057 TTACTGATCCTCCGCTGTTTCTTCATCTGTAAAGTGGAGTTAATGACAGACAGCTGGATG 72915116

72915117 TCAACGTTTTTTGTTGTT

C9-OFF1：

>chromosome:GRCh38:13:36202761:36203378:-1

36203378 ATAGAAAACGTATTTTTATGAGAATAAATCTATAACCGCCTGGAAAATGAGAATGTTCCC 36203319

36203318 AGTGAATCAGAAATTTTGAGGAATTGTTGAACTATATTGTTAAATGAAAAAAGCAACTCA 36203259

36203258 CTTCAAAAAATACTAACAGCCCATTTATGAAAAGCTATGTATGTGTGTATCGTACTTATA 36203199

36203198 TGTTTGACACAAAAATCATCTGGAAGGATAGACACCAGACTCTTGACAGCAGTTCCTTTC 36203139

36203138 AGAGAAGGGATTGAATTAGGATATGAGAGATGGTAAAGGAAGGTTGACTATTTTA**TAAAA** 36203079

36203078 **AAAAGGAAGAAAATTAGG**TGAGCAGGTGGAAGTGCCAAAGCTTTGCTTACTTTACTATTG 36203019

36203018 CCTAGTTTCTTGCACCTCACCTGTTCTTTCTTGCAACCCTCAGGAAACCAGGCTGAAAAG 36202959

36202958 AGAAGGAAGCCCCACGGGGCCACTTACACCAGCATCCTGCCCTGTGGAGTTGCTTCTTTG 36202899

36202898 CCAAGCTGCCTTTCTCTTTGTACAAGTATTTCCAGCCCCTTCCCTCACTCTTAGCTTCAA 36202839

36202838 CTCTAATCACCTCCCAAATCTGTTTTCTCATACAGATGTCTATGGCCTGGCATTTCCAAT 36202779

36202778 TGTCTTCTATTTCCATTA

C9-OFF2

>chromosome:GRCh38:7:51656757:51657374:1

51656757 TAAGTGCCTGTTCAAGGCCCTCCACCTGGTCTCTCTGGCCTTGGGGCTCAGCCGAAGTCA 51656816

51656817 GGGTCCCCTGTGACTGCTCCCACTGGGTTAGCACAGCCTCCAGACTCTGGGTCCCATGTA 51656876

51656877 AGGCCATGTAGCCTGACTCCAACCTGGCAAGCTTAGCAATAGAGAATGCATCTTTTAGGT 51656936

51656937 CATGTGTTATATTAATTTTATAAAAGCTTGAATTTTAGAATAAAAAATAGGCAGAAAACT 51656996

51656997 TGGAATCAGTACAAGGAACTATCAGAAAGCTTATAGTATACTGAGGATTAAAATG**AGAAA** 51657056

51657057 **AAAAGGAAGAAAATTTGG**CCATGACAGTAAAACAGGGGTGGGGGAGGCTTTAAACTTGCT 51657116

51657117 TAATGACCAGAAAGGACAGAAATTAAAAGAAGAGAGTGGAGGCTGGTTTCCAGGGAACAA 51657176

51657177 ACCATATAATAAGATGATTGGAACAGTCAGTCTTGCCCTCTACCTTCAGGGAGGGGAGAG 51657236

51657237 GGGCTGGAGATGGACATAGTCACCAGTGGCCATGGATTTCATCAATCGTGCCTATGCCAT 51657296

51657297 GCAGCCTTCACAAAATCCCTTAACCACGGGGTTTGGAGAGCTTCTGGCTGGTGAACAAGA 51657356

51657357 ATGCATCTTTGAGCCAGG

C9-**OFF3**

>chromosome:GRCh38:5:147662712:147663329:1

147662712 CAGTGCCTCATGTCTGTAATCCCAGCACTTTGGGAGGCCGAGGCGGATGGATCACCTGAG 147662771

147662772 GTCAGGAGATCGAGACTAGCCTGGCCAACATGGTGAAACCCCGTCTCTATTAAAAATACA 147662831

147662832 AAAATTGGCTGGGCGTGGTGGCAGGCACCTGTAGTCCCAGATACTTGGGAAGGCTGAGGC 147662891

147662892 AGGAGAATCTCTTGAACCCAGGAGGCAGAGGTTGCAGTGAGCCAAGATTGCACCATTGCA 147662951

147662952 CTCCAGCCTGGGAGACAGAGTGAGATTCTGTCTCAAAAAAGAAGAAAAAAAAAA**GAAGAG** 147663011

147663012 **AAAAGGAAGAAAATTGGG**GAGTCCCAGGAGTAATAGCTAGTTGAGTGATATCGAGTCTCC 147663071

147663072 ACCATCTGTAAAATTGGAAGGTTTTACAAAATAATAGTTAAGAACCTTTCCAGTTGAAAA 147663131

147663132 ATCTTAAGATTGAGACTCCATATTTTAACCACTGTAATATAAAGTGTCTGTGATGGCTAA 147663191

147663192 TACTGAGTGTCAACTTGATTGGATTGAAAGATGAAAAGTATTGTTTCTGAGTGTGTTTGT 147663251

147663252 GAGGGTGTTGCTAAAGGAGATTAACATTTGAGTCAGTGGACTGGGAAAGGCAGACCCACC 147663311

147663312 ATCAGTCTAGGTGGGCAC

C9-OFF4

>chromosome:GRCh38:2:158230685:158231302:1

158230685 CGATATGAAGTGGAGCACGGGTAGTAGAGAAGCTAAGGAACAACAGGGAGACCAGTTAGA 158230744

158230745 AGGAAAAGGCAGCGCTCGTCCCTGGACTCAGGAGATGGATTCAAGTGTTCAGATCTGAGA 158230804

158230805 TGTGCTTTTGAGTAGAATCAGAGGGCTCATTGAAAGATTCCAAGAGGGCTCATTGAGAGA 158230864

158230865 TTCCATGTGAAGATTGCAGGAAAGACTAATCAAGGACAATTCCTAGGTTTTTGCTTGAAC 158230924

158230925 AGCTGGTTGAAAGGTGATACTAGTAACTGAGATAGAAGATTGGGAGAATAACAAG**CTTGG** 158230984

158230985 **AAAAGGAAGAAAATTGGG**AGCTCAGTTTTGAACACATAACACTTGACAAGCCCATAAGAC 158231044

158231045 CTCTAAGTTGGAGGGGCCAGACGAACATCTGAAGGCACAGATGGGTGGCTTAGGGGAGAA 158231104

158231105 CTCAGTGCTGGAGACCTAAATTAAGGAGATAGCCACATAAACAGTATTTGACACCAGAGG 158231164

158231165 ACTGGATGAGGTCTCCAGGATGAGGATGTGAACAGGAAAGAGAATGTGGTTATGCACTCT 158231224

158231225 CATGCTTATTTGCTTTTCTGCACAGCTTTCTCTACTAGAATGACTTAATTTCCCTCTTCC 158231284

158231285 ACCTGTCAAACTTACTTT

C9-OFF5

>chromosome:GRCh38:11:79918132:79918749:1

79918132 TTTAAAGACACTGTCTCCAAATACAGTCACGTTCTCAGATACAGGGACTCAGGGTTTCAA 79918191

79918192 CACAGGAAGTTTGAAGGGGGCACAATTTAATCCGTAACACTATTAATTTAAAGCTCAATG 79918251

79918252 CTTAGGTCTGGAATAGTCAATTAGAGGAGAGCCCAAGCTCCTTGACACTCAAGACCATGC 79918311

79918312 AAGCACTGCAGCAGTGGCCACTGGACACAAACACAGACTGTTCATGGAAAGCCTGACACC 79918371

79918372 CACCATGGCCTTGTCATGCCTTGGGGCTCAAAGGAACCTGGGGAGGCACAGGCTT**TTGGG** 79918431

79918432 **AAAAGGAAGAAAATTCGG**ACAGGGGCCTACCTCACTCTATTTCAGAACTTGGATAGGATG 79918491

79918492 CATATTCACCACCCACTGCTCAAATAATGGGGCCAATTTGGGTGGGACTTGAGTCTTTGC 79918551

79918552 TTATACCCAGAAAGGTTGAAAATGGTTGGTGGGATACTGTGTTTGGTTAGACTTACAGCC 79918611

79918612 AGGTGAAGAATGGCCTTGTGTCTAAAACAATCACTGCACTGGAAGGAGGCAATTATAAAA 79918671

79918672 TTCCCTGAACGTTTATTTGCTTGCTGTTTCTTAAGCATAAGCAAACTGAAAAGACACCTA 79918731

79918732 TTCTAGAGTTAGGAGTTG

C9-OFF6

>chromosome:GRCh38:4:131198819:131199441:-1

131199441 AAACATCATGTGATACATAATGAAACCACAATATCACCTGTCAAATAAATACTAAAATAA 131199382

131199381 AAAATAAAAATAAACCCATTTACTTTCACCTTAGCATCCACGCTATTAACCAATACAACA 131199322

131199321 ATTTGCCTATAACCCAAATTCCACAGAAAGACCAGAAATAGAGAAAACCTAAAAAAAAAA 131199262

131199261 AATGATTGAAAGAGTGAATTTTCAAACCTTAAGAGAAAGATATATATAGAGAGAATCAAA 131199202

131199201 TACATGCTTCTAAGGATAGGAAGTAAAAGCAGAAGGTATATACATGTTTAAGGAATTTGA 131199142

131199141 **GGAAAAAAAGGAAGAAAATTAGG**TCACAACCACAAGTAAAACATGAATTAAAGCTATAAT 131199082

131199081 GTAGTAGGATAATTTTTCTACTTTCCTTATTACAAATCCATTATTTTTGATTAATGACTT 131199022

131199021 TTTAAGAATGTCTCTTTACTTTTGGCCACAGATTGAGGTCAGTGGTATGAGGAATAACAA 131198962

131198961 GCAAAACACAGAATTATGTTTACAAGTAGAAATCTAGTAACTGTAGACTTGTAAAGCACA 131198902

131198901 TATTTTACATTGTGTCACAGAGTTCTGTTTCAATTTTGTTAATTATATAAGGAAAATAAA 131198842

131198841 ATAGGTTCCCATAAACATAAACT

C9-OFF7

>chromosome:GRCh38:1:25887281:25887903:1

25887281 TTAAACAATGACAAAAAGGGGTACAGGAAAAGGGGCCAAGAGGTTGTCCAGGAGCCTGCA 25887340

25887341 GCATTTGCCCATAGTTACGGTAAGTCTACCCCTCAATCCCGAAGCTTTCCTCAGTGGGTT 25887400

25887401 AACAGGAAAGCCGGTGATGCTGAAACAAAGTGGGGAATGGAGTACAAGGGCTATCTGGCA 25887460

25887461 TCTTAGATGGCTACATGAACATGCAGCTTGCAAATAAGAGAAATACATAGGTGGGGAGCT 25887520

25887521 GTCTGGACATCTGGGTGAAGTTTTAATATGGTATAAAAATGTCCTTTATAGTAGAGGTAT 25887580

25887581 **GGAAGAAAAGGAAGAAAATGGGG**GAAGTGAGAGAACAGCATCTTTTCTGGGGGATTTTGA 25887640

25887641 AAATATAGATTTCTAGACAATAAAGATGTGTTTTTTGGGGTTTTTTTGTTTTGTTTTGTT 25887700

25887701 TTTTTGAGATGGAGTCTCGCTCTGTCGCCAGGCTGGACTGTAATGGCGCAATCTTGGCTC 25887760

25887761 ACTGTAACCTCCGCCTCCCGAGTTCAAGTGGTTCTCCTGCGTCAGCCTCCTGAGTAGCTG 25887820

25887821 GGACTACAGGCATGTGCCATCATGCCTGGCTAATTTTTGTATTTTTAATAGAGACGGGGT 25887880

25887881 TTCGCCATTTTGGCCAGGATGGT

C9-OFF8

>chromosome:GRCh38:12:79298113:79298735:-1

79298735 CCTTAGGATGAGTGGTGGCAAAAGGCCAAGAACTGATGAATTCTCCAGCTGTTCAAATAT 79298676

79298675 ATCTGGGACAGATGCTATGGGTTGGGATGTTTATTAGTGACATTTTTAATATTCAGTGAA 79298616

79298615 AGCAGTGCAATCCAAGTAAGCTACACTTCAGTAACTGGGTAGAAATACCTAATTAAGAAA 79298556

79298555 TTGTTTAGACTTGTCTTAAAGGTTTGATCAATTTTCATACACTTCCTATTATTTTATCAG 79298496

79298495 TGTAAATGGTTGTCTTATGCTTCAGCAAACATTTCTCAATTAACTCTGTATATTAAGGAG 79298436

79298435 **GGAAGAAAAGGAAGAATATTGGG**CCATCTACATACAAGAAATTTTAGTTTTCCAAAGAGT 79298376

79298375 TTTTCTTTTCTGTCATTTATTTACCATCTGTTTGGAACCTGATTATTTTATTAAAATGAT 79298316

79298315 ATGATTAAAAAGTAAGCATCATCCTTTTTCCATTTTAGAAGGAATGGAAAATAAACCCTT 79298256

79298255 CCAAAGAGCTGACTCTCAAAGAAAGAAACAAAATTTTTTAGAGTAAGGATAAACCCTTGA 79298196

79298195 AAATGTTATTGTATATCTAAAATCTCTCTAGACTTTCTAGTGATTGTACTCAAAGAGCAA 79298136

79298135 CAGTCAAAAACCTGGACTTGGGG

Tale-OFF1

>chromosome:GRCh38:X:86925532:86926146:1

86925532 CTCAAGTGATCCACCCACCTAGGCCTTTCAAAGTGTTGGGATTACAGGCATGAGCAACAG 86925591

86925592 CACATTTTTTTTTTTTGAGGTTTTTAATGGTCAATGTAGTTCTCACTTCACTTGTCATTT 86925651

86925652 TTCCTCACAGAATCCTTTTTGATTGGCACATATACAACTTAATGTAACACATAGCAAAAC 86925711

86925712 ATCTTAGCATACTTAGTGGTACACTAGAGGCCAGAAAGTGTTAAAGGATAAGGCCTACAT 86925771

86925772 TATAGTTAGAATTTTTTTATAACTCTTTCTATTTAATATTTCTCATCTTTTAACTTCGTT 86925831

86925832 **GATAAAGATAAGCAG**TGGATTCAGGGGAGAATATTCAATGATTTCTGGCCACAGTCTGGT 86925891

86925892 TGAGTAGTACACTGAGGGTGTATGAGGAAATGAATCTAGTTGTGAAGTAGTTATGAAGCA 86925951

86925952 GTACTAAAATCATCAATCAAATCTTATACATACAATGTTTGTGGGATACCAGTGGAGGAA 86926011

86926012 CTATAGGAGCTCTGTACATAGTCTTGGTGGCCTTGAATAGTCAAGATAATTAAAAATGAG 86926071

86926072 TTGAAAAGTGTTCTTCATATACTTTAAATTTGAACTGCAGCCTGTATATTAGTGATACTT 86926131

86926132 TATATGAATATGATT

Tale-OFF2

>chromosome:GRCh38:20:49280500:49281114:-1

49281114 AACAAAGACCTAGTAATACCAACAGGCCAATGTCAGTCCTAATGACTTATTCTCCCAGTT 49281055

49281054 TTATACCCAACTTGGAAATGGGTCCTGAGGTTAGGCCCAGTTGGACAAATTTTGCGGTGG 49280995

49280994 GGTTTGGCAGATAGAAAGCAGAAACCTCACCTGTTACAAGGCAGACTGAATCAAGCCAAG 49280935

49280934 ATCAACACACACTGGTACACGTGGCTCCCAACCTATAGAAAGTTAACAGGTCAAAAGAAG 49280875

49280874 CCATCTGAATGCTGGAAAAAATAGAGAAAGCCCAGTTCCCATGAACAAGCAATAGTCTCT 49280815

49280814 **GATAAAGATAAGCAG**AAAACAAAGATAGGAGTTATTACTGGGGCCAATGCATCAGACAAA 49280755

49280754 ACTGGCCAATAGTGTAGAGATCAACAGGGTCGATCTGATGGGGAAAGAAGCTGTATCATC 49280695

49280694 AGCTGCAAAGGCCACATTTGAGTCGTACAAGAAGTAGGGTGACAAACCCACACTGAAAAG 49280635

49280634 CAGCTACTACCCCCTCCAGAGCCTGTGTCTACTCAGCCAGGGAAGACGTGTTGGCACCAC 49280575

49280574 TTACCAATTTTATATGTATATATATATTCTACTTCCAACACCCGCATTCATCCTGGTTCA 49280515

49280514 ATCAAAGCCTGGTTT

Tale-OFF3

>chromosome:GRCh38:18:25405589:25406203:1

25405589 TCCCACACAGCAAGTAACTCTGCAGTGAATGCCAGCTCGGTGTCCTCCGATTCAGTTCTG 25405648

25405649 GCACTATTTACCTGCGGATAGTGTCAGATCCCACAGGTTGAAGGCTCGGTCCCACAAGAC 25405708

25405709 TGCCCCCGACTTCAGATGCTAATTGCAAATAGCAGGTTGTCACCTATGCTTCTGACTGGC 25405768

25405769 TATAAATTGGGGTTTCCATGATCCCCTCCTTGGGTTTGATTAATTTGCTATAGCAGCTCA 25405828

25405829 CAGAACTCAGGGAAACACTTTACTTAGGTTTGCCCATATATTACAAAGAATATTTTAAAA 25405888

25405889 **GATAAAGATAAGCAG**CCCTATGGAAGAGATGCATAGGGCGAGCTATGGGAGAAGCGGTGA 25405948

25405949 GGAACTTCCATGCCCCCTCCAGGTGGGCAACCCTACAGGAACCTCTACATGTTTAACTAT 25406008

25406009 CAGGAAGCTCTCTGAACTCAGTTATTTTGGGTTTTTATGCAAACTTCATTACGTAGGCAT 25406068

25406069 GATTGATTACATCATTGGCCATAGGTAAGCCATTCAACCTTCAGCCCCTCTCTCCTCCGT 25406128

25406129 GGAGGTGAGGAGTGTGGCTGAAAGTCCCAATCCTCTAATTATGCCTTGGTTTTCTGGGAA 25406188

25406189 TGAGCCCCCATTATG

Tale-OFF4

>chromosome:GRCh38:18:37513444:37514058:-1

37514058 GAGCATCTGCGTTTGCTGGACTTGGTAGAGCCATCACCTCAGAAGTCACCATCCCACACC 37513999

37513998 TCTGTCAGAAGGTATACACACACACACACACACACACACACACACACACACACACACGGC 37513939

37513938 ACCACACAGAAGGGAAGAGGGACAAAATTAGGGCCTCATGGACCCATCAGAGGACAACAC 37513879

37513878 AAGAGAGGTAGTTAGGACTGTAACTGCTGGAGCAAGACAAGGCATGCAGAAGTTCATGAA 37513819

37513818 CCCTTCAGGGAGGCTCCATAGAGGAGGGAGCACTGCGGATTGGCCTGAAAGTACAGGTTG 37513759

37513758 **GATAAAGATAAGCAG**AAAGTGCTAGGGAGGGTATTCCAGGAGCTGGAACCAGGATGAGCA 37513699

37513698 TGGACAATCTGGTAGGAATGAGCAGGTGTGTCTCAGGAACAGTAAGAAGATGTTCTTGAC 37513639

37513638 AGAAGCTTAGTAGGTAGAGCTGGAAAGAGAAGGTTGGTGCAGACTAAAGAGGCCCTGGAG 37513579

37513578 TGAGTGAAAGAATGGAGACATCAAATCCATTCCATAAGAATGGTCCACGCCCCTGCCCAG 37513519

37513518 TAGCTCTCCATGCCTTTCTCTTTGGAAAGTAAAGCTCAGACCAGAGGAAACCCATATTGC 37513459

37513458 TGCCAATCCATGGAC

Tale-OFF5

>chromosome:GRCh38:13:83554776:83555390:-1

83555390 TTTTTAAAATTTAGTCTATCCTAAAGCACATATACAATATCATAAGACTGATACATGTGT 83555331

83555330 AATAAATGCTAATGAAAAGTAATTACTAATTGATGTTTTCAAACGTGATTGACTAATTAG 83555271

83555270 TACAGAATAACCCTCCTGATGTTATACTTACAACATTTGACAAATATATGTAACAACTAT 83555211

83555210 TTGCATGTACTGTATGATAAACAATGCAAGACCCTCATCCTTGAAAAGGGTAAATATATA 83555151

83555150 AAGAAATCTACATTTTCTCTGCCTTCTGTCTGAGAAAAATCTCCTTTCATAGCACAGTGA 83555091

83555090 **GATAAAGATAAGCAG**AACTTTCAGCTCGCAGTAGCCTGAGGAAGCAGGTTTCTTCCAAAG 83555031

83555030 TTGGATTTCAGGATCAGTTTCCTAAAGAAAAAAGAGCTGAGCAGAGCAGGTGATCAACAA 83554971

83554970 TTTTTGTGGAGGTTCATTATGGTTCTTCAGACGAGACTAGGCTGCACATGCACGGGGAGA 83554911

83554910 CTCTCTGAGGCCCAGTAGATTCTAATTGTTGCATGACTGAGAAGCTAATGTTGATAGAAA 83554851

83554850 GTTATTCAGGGCTGAAGCGTGTTGGAATCTCAGCCTAGCCTAGCGAGAAGAAACATATCC 83554791

83554790 CACTGAGCTCTGGTC

Tale-OFF6

>chromosome:GRCh38:1:116125931:116126545:-1

116126545 CTCAGTCTCTTAGATGGTATTTGTTTTTACCATCCATTCAGCCAGCCGAGCCTCAGAGGT 116126486

116126485 ATCTATCATCAGAAACCCCTGTAACCACAAAATACATCAAAGCACGTCACAGCCACTGTT 116126426

116126425 TCAGAGGATTGAGTGATTTGAAAGAAGTCTCCATTGCCTCGTTTTCCAACCAGGCAAAGA 116126366

116126365 GAAGAAGAGAAAACTAACGCATCTAGTAGTTTTCCAGCTCACATACTATAATTTCCTCAT 116126306

116126305 TTCCAATTTGCTCCTTCTTCTTTCAAAAGGTACAGTTTTTGTTTGTTTGCCTTCATTGCA 116126246

116126245 **GATAAAGATAAGCAG**TCTTGAATAGATCAGGATCTATTCATCCTTATTTGCTCATCCCAA 116126186

116126185 TTCTTGAAATAGATTGGAATCTATTATTTTGCATTTCCTTGCACAGAGAAGAAAATGGAA 116126126

116126125 GCACATTAATTGTGTTAGGCTGTTCAAAAGGGAAACATTCCCTGGCCAGGGTGGGATAAA 116126066

116126065 GGGCTTGTCTGCATCTCATCAGAGCTGCCCAGACAGGAACATGACAACTGGCACTTTAGC 116126006

116126005 ATTATTTACTAAAAGCGCCTCATAAACCTTGCCCATTCTGAATCTCTGCTTTGCACTTTC 116125946

116125945 ACCATGAATAATGTC

Tale-OFF7

>chromosome:GRCh38:1:66528676:66529290:-1

66529290 TATATATGTGTGTGTGTGTGTGTGTGTATACTATATATATATACACACAAGTTTCTATGG 66529231

66529230 CAAACAGAACCCATAATATGATTGTATCCAGAGAGAAACCAGCAGAACCTGTAGTCATGC 66529171

66529170 TCCTGTTTCCCTGGGGGGAAGACTCTATGTGCTGGCCCACTTATGCCAAAGACAAGACAT 66529111

66529110 GCCCTTGTATGCTTTTCACCCATTGGACTATGAGCATCCCAAGAGCCATGTGTTCATAGC 66529051

66529050 TACATGAGTGCTGAAGGAATTTATATCAGCTGGGAGTACTGCTTAATCTAGACCTACATT 66528991

66528990 **GATAAAGATAAGCAG**TAACACTGAATCTATGAGAAAATTCAACAGTTTCAAAAGAGAAGG 66528931

66528930 AAAATAACTACAGCTCACTGAAGCATAGGAAATGTCCCCACCGAGGCAGAGGAAATGTGG 66528871

66528870 AGAACTTGAGAGGCATTTTTCAAAAAATCTGTTTTTTAGTTTTGATATAATTTCAGAGAT 66528811

66528810 ACAGCAGCAATAAATAAGAGCAGATTGCTGTGATAAAAGAGCATACTGAGAGCCAGAAAG 66528751

66528750 AGTGCATGGAAGTAATAAAAACATTCTCATTAAACATTTAAAAATGTTATAGCTATAAGC 66528691

66528690 AGTGATATAATGGGT

Tale-OFF8

>chromosome:GRCh38:11:30658149:30658763:1

30658149 CACACCAATCAATTATTTAGCAGATTCTAGCTGGGCACCCTTTAATTCAAGTCAGTTATG 30658208

30658209 ACACCACATACCCGGAAATAGCATTAGGTCCCATGGGTTGAGTTCAGTTCCATGACTGGG 30658268

30658269 CCGCTCCAGACGCCATCCACAAGCCCCCAGTTGTCATCTGTTCTTCTGACCAACCAGCTG 30658328

30658329 TAAATCAGGGTTTCCACAATCCCCTTCCTCAGGTTCCACTAATTTGCTAGAGGGCCTCAA 30658388

30658389 ATAACTTAGGGAGACATTTTACTTATGTTTGCTGGTTTATTAATAAAGACTATAATAAAG 30658448

30658449 **GATAAAGATAAGCAG**AGATACATTGGGTGAGTCTGGAAGGGTCCTGAGAGCAGGCATTTC 30658508

30658509 TGTTCCCATGGAGTTGGGATGTGCCATCCTCCCAGCCCAGGGATGTGTTCACCAACCTGG 30658568

30658569 AGGCTGTCTGAACCCCATAGTGCAGGATTAATCACATAGGCATTATCAATTATTAACTAT 30658628

30658629 TTACACCCCATCTCTTCTCTCTGAAGAATGCGAGGGTGGGGCCAAAATCTCCAAGCTTAT 30658688

30658689 TATCATTGCTTGGTCTTTTTAGTGACCAGCCCTCAGCCAGGTGCCCACCAAGAGTCACCT 30658748

30658749 CATTACAACAAAAGA

Tale-OFF9

>chromosome:GRCh38:6:153727907:153728521:1

153727907 CCTTTGCTAAAAGAGGCTTTGTAGTCCTGACATATTACTTACCTTAAAAGCAGCAGCAGC 153727966

153727967 ATAGATATTCAGCCAGCCAGTGCTAGAAATTTCTCATGACCTGGTTATCTTACTAACAGT 153728026

153728027 TTGTATTTTAGTGAGTACACTGTCGGATACAGCATCCACTGCTGCTGTTTTCATTTTCCA 153728086

153728087 GCACAGAACTACTGTGCTTTGTTTGTCAAGAATTCTTTCCTGTTGAGATAAAGCCCTGAG 153728146

153728147 GCACACATTCAACATGGATGAGTCTTTTGGTACTTTAAGCTGGAGGCCTAATTTTCACAA 153728206

153728207 **GATAAAGATAAGCAG**GGTTGTAAAAAAAAAATCCATCAAGTATAAACAAGAAATCATATG 153728266

153728267 CAGACTTTCAAAAATTATTTTTAAATTTCCTGAATTTGTATAGTTTTAAACTTTACCTCA 153728326

153728327 ATATGACACTCATGCAAGTTTTCAAAAATTGAATCAGCAATTCTTCACATCGCTATAGTG 153728386

153728387 TGACATTTTATGAAATATCTGTCTCCCTTTTCTGCAATGATTCACGTAATATGGGAAATA 153728446

153728447 TTATGGTGATATGAAATTTCTTTGATTTTTTCTAGCAATTTGCCAAGCCATACATGTGCT 153728506

153728507 TTCACTATCAGTACA

Tale-OFF10

>chromosome:GRCh38:2:1806244:1806858:-1

1806858 TCCTTAGACAATTGATCCGAGTCCACGTGCCTTTGTTCCCCATTAATGAAGGAAGTGGCG 1806799

1806798 GTTAGGTTAGGTAGGCCAGTTCAAAACAGAAAAGGAAAGAAAATACGGAGAGAACAGATC 1806739

1806738 TGCCAATCCTAGAGGAGGGGCCATTCACACCTGGGCTGGTCCACCTGGGCGGCGGTGGGC 1806679

1806678 AGATCCCTGAGGTGGCCATCTCTTCATCGGTGGGCCTGAGTCGGCATTGAGGGCACTAGA 1806619

1806618 CATTCCTATTACTGCTGCACACAAGGAGTTAATTCACAAAAACAGGTGCTAACTCGGAGG 1806559

1806558 **GATAAAGATAAGCAG**TTGGTCTGTGACTCTAAACTAGAGGAACCAACAACTGGGTGTCAT 1806499

1806498 CCCACCAGAAAGCAGCCGGGACACGGGCATTCCAGGGCCACCGTCCGCCCGCTGGGCCCA 1806439

1806438 GCTGCTGCTGCTGGGCAGCCTTCAGCCAGGTCGCTGCATTTCACCCAAGTTTCTAGACAC 1806379

1806378 ACAGTTCGTGTTGTGCTAAGTTTTAGGACAAACAGTTCCCCTTGCTGCAGATCCTAATGC 1806319

1806318 ACGCACGTCCATGGGTAAAAAAGAGGAGCTGCAGGTCACTGGGAGAACAGGTGGTGGAAG 1806259

1806258 TGGGTCTTCCTAAGT

Tale-OFF11

>chromosome:GRCh38:2:43161480:43162094:-1

43162094 AGCCGGGGCATTGCTGCTGGGTGCTGAGGACAGGGCTCAGGAGACCTGGGTCCCCGGCCA 43162035

43162034 GCTCACCCTGGGGCTGTGCATCGCTTTTCTCCCCAGCCAATGGGAGAAGGACTCTGTGCT 43161975

43161974 GTCTTCCTGGTGAGGATGGTAAGGATCACACCAAGGACTCTGCAGGGAAGGTCCTGAATG 43161915

43161914 GATCAGGCTGCTATCTTGAGGAAAGAGGGTTATTATTCACAGTAGTCATAAATGCCCTGT 43161855

43161854 GAAATTGGAGTGGAGTTGCTTTGAGCTTTGGCCAGAATGCCCCAATCAACCTTTGGCTCT 43161795

43161794 **GATAAAGATAAGCAG**GCTCAAGGTGATAACACATAAACCAGAAACTGTCAACAGGAGCTA 43161735

43161734 ATTAGGGCACTGGGTGCCGGGGTGAGCCACGCCCCCTACACCTGATGGAGGAGAAATCTC 43161675

43161674 AGGTGGGGGAAGATGTGGGCTCATTGCCTGGACCTTAGCCTATTGCTTTTTTGTCACCTT 43161615

43161614 CTGGCCACTTTTCCACCTCCAACCGCTACCTCCCACCCCTCTGCCTGCATTATATAACAT 43161555

43161554 AACATGAAACAAGATGGAATAAAACAGAATCCAATGTGATGGGTGCCATATAACACAGTG 43161495

43161494 GAAAGAGTCCTGACT

1. DYRK1A#1

| TaC9 target locus | On target sites | gRNA off target sites | Tale off target sites |
| --- | --- | --- | --- |
| DYRK1A#1 | 1 | 11 | 2 |

On target：GTTCCCATCACCATCACCACCACCACCACCATCACCACCACCATGG

>chromosome:GRCh38:21:37511781:37512398:1

37511781 ATTAAAAGTTTCAAAAACCGTTTCCCCCTGATTAATATGATGCTAGTTTGTTAGTGTCAT 37511840

37511841 GGCTTTTGATGGAAGATTAAAGCTTGGAAACAGTCCTCTGAAAAATCCTTTTAAAAATCT 37511900

37511901 GTTCTTTCAGGTGCGTCAGCAATTTCCTGCTCCTCTTGGTTGGTCAGGCACTGAAGCTCC 37511960

37511961 TACACAGGTCACTGTTGAAACTCATCCTGTTCAAGAAACAACCTTTCATGTAGCCCCTCA 37512020

37512021 ACAGAATGCATTGCATCATCACCATGGTAACA**GTTCCCATCACCATCAC**CACCAC**CACCA** 37512080

37512081 **CCATCACCACCACCATGG**ACAACAAGCCTTGGGTAACCGGACCAGGCCAAGGGTCTACAA 37512140

37512141 TTCTCCAACGAATAGCTCCTCTACCCAAGATTCTATGGAGGTTGGCCACAGTCACCACTC 37512200

37512201 CATGACATCCCTGTCTTCCTCAACGACTTCTTCCTCGACATCTTCCTCCTCTACTGGTAA 37512260

37512261 CCAAGGCAATCAGGCCTACCAGAATCGCCCAGTGGCTGCTAATACCTTGGACTTTGGACA 37512320

37512321 GAATGGAGCTATGGACGTTAATTTGACCGTCTACTCCAATCCCCGCCAAGAGACTGGCAT 37512380

37512381 AGCTGGACATCCAACATA

C9-OFF1

>chromosome:GRCh38:3:137961552:137962169:-1

137962169 GTGGCTATTGTGTGCTGACTGCAATTACAGCCCTCATTTAAAAATGATTAAGTGGTTATA 137962110

137962109 ATTATTTTTCAATTAAAACTTATATCTTCCATTTATGCAACATTTCAGACTTCAATTAAA 137962050

137962049 CTTCTTTTTAAAAGTGCCACTGAACTAAAAGATTCCTGTTGCCTCTTGCCAGGAAACCCT 137961990

137961989 AAACTCAATCCAGATAGGATTTTGATTTCCAGCCTTTGGAATTCGTTATATTAAATTTAG 137961930

137961929 ACATTGTGATGAAAGTTAACAAGCAATAAAATTTGCAGTGCCTGCTGGAAGTGAT**CACCA** 137961870

137961869 **CCATCACCACCACCATGG**GTGCGGAGGAGAGAGACAGTTCCAAGCTCTTCTTGGGGAGGC 137961810

137961809 TCGAGAAATTTGTGACTACTGAGAAGAGAAAGGTGTCCTTGTCATCGTAAGGTAGTGTTA 137961750

137961749 CTAGAAAAGCTGCTGCCTGAGGCCCAATATTTAGAGCAGCAACCTCAGCTGTGGTCTTGT 137961690

137961689 TACAGTAATGAGTTACTGTAAGGAAAGTGTGACATTTCGAGCAATTTGATTTGTTTAAAA 137961630

137961629 ACTAGAGCAGTTTCAGGGTTTTCCTTGTAAATCTGTCTTATGTGTCTTCAATGTTCTTTC 137961570

137961569 TTGAGGAGTAGAGAAAGG

C9-OFF2

>chromosome:GRCh38:3:49318633:49319250:1

49318633 GTGTGGTGGCTCACGCCTGTAATCCCAGCACTCTGGGACGCCAAGGCGGGTGGATCACCT 49318692

49318693 GAGGCCAGGAGTTCGAGACCAGCCTGGCCAACGTGGTGAAATCCCATCTCTACTAAAAAT 49318752

49318753 ACAAAAAATTAGCCGGGCGTGGTGGCACGAACCCGTAATCCCAGCTGCTCAGGAGGCTGA 49318812

49318813 GGCAGGAGAATCGCTTGAACCTGAGAGGCAGAGGTTGCAGTGAGCCGAGATCGTGCCACT 49318872

49318873 GCACTCCAGCCTGGGTGACAGAGCAAGACTCTGCCTCAAAAAAAAAAAAAAAACA**AACCA** 49318932

49318933 **CCATCACCACCACCAAGG**AATCCATCCATGGAAACATTTACTATCCTCTGCCAAGAAATA 49318992

49318993 TATTAAATTGGAGGGAGGACTGTTGAGGGATAAATAAATAAATAGAAATACATTGGCCAG 49319052

49319053 GCATGGTGGCTCACACCTGTAATTCCACCACCTTGGGAGGGATTGCGCCACTGCACTCCT 49319112

49319113 GCTTGGGTGACAGAGCAAGACTGCATCTCAAAAAAAAAAAAAAAGAAATATATAAAACTG 49319172

49319173 ACTCTATTGGTAGGTTTTAAAAAAGTTATTAAGCCAACTAGTGACCTTCCTTCGATGACA 49319232

49319233 TATTCCCTTGATGTACGT

C9-OFF3

>chromosome:GRCh38:16:80170909:80171526:1

80170909 ACTGCTAATAGATACTCACAGTTAAAAATAAATTTAAAAAGAAATAAAAAGGAAGGACTG 80170968

80170969 AGGCTGCAGCCCTTGCTATCTCTCTTTATTGCAGACCTTCTTTTCTTTCTTGCTGCCTCC 80171028

80171029 ATCTGTGATATGTGTGGGTAACACAGGTTAGTGGGAGATGAAGTTTAAAGAATAGAAAAT 80171088

80171089 TATCAACTTGACAACTTTCAGTTGAATGAAGATAATAGTTCAGTTCATACTTTAAAGTGT 80171148

80171149 AGGGTAAGTCCACCATCATCACTACCATCACAGTCATCTCCCCCAGGTCTATGAC**TACTA** 80171208

80171209 **CCATCACCACCACCAGGG**CTACCACCACTATGACCATCACCACCACAGCACCACCATAAT 80171268

80171269 CACCAGTACCACCATTATCATCACTACTGCCAATGCCATCACCATCACTACATCCGTGCT 80171328

80171329 ACCACCACCTTCACCATTACCATCTCATAACTACCAACATCACCACCACCACCATCACTA 80171388

80171389 CCACCAACCATACAATCACCATCATCATCATCATTACCACCAGAGATACCACCACTACCA 80171448

80171449 TCACCATCACTGTCCCGCCACTACCACTATCATCATCACCACCACCAATACCAACACCAA 80171508

80171509 GACCATCATCACCACCAG

C9-OFF4

>chromosome:GRCh38:2:23510141:23510758:-1

23510758 TCCAGCCACCACTTGTCTCCCTTAGGGCAGGAGCCGCCCTCCCTCTGGAGCTCTAACCTC 23510699

23510698 TTGTCATTGTCACCAGCCACTCACTAAGCTCTGCTCTCTGGGCCAGGTGCCACCCAGCTC 23510639

23510638 CTGCTCCCTCCCTCCCAGATAAGCCAGGCCTTATTGTTTGTAAGACTTGGCTGGTGACAA 23510579

23510578 TCGGGGGACCCTGTGGGCAGACCCTCAGGAAGGGGGCAGCTCTGCTTGTCCCAGCCTGTG 23510519

23510518 TTTAAGGGATGGACAGACCCACAGCAAAGCCTGTTGAGGCTTGCTCACAGGGACA**GCTCA** 23510459

23510458 **CCATCACCACCACCATGG**CTGGGGTGGCCCCACACAGCAGGCCATAGAAGGGTCAGGTGC 23510399

23510398 TCACAACTGGGGCTGAGCTAAGGGAGGGTGTCCTGGGATGCCAGCCCTAGAAACTGGATA 23510339

23510338 CTAAATGAGGAGGTGGCTCAGCAGCAAACCTCAGCGGCCCTGGTGCCCCCAGTCATGCTT 23510279

23510278 CTTGGTTAAGTCTCCCCCATCTTCCAGACATTTCCTGTCACTCCCCAATCCATCCCCTGC 23510219

23510218 CATAACCACTAGAAATAAGCTCTTCTGAGGGCAAGATTTCAGCAAACTCCCCTGTCACTG 23510159

23510158 AGCACATGGCCTCACACT

C9-OFF5

>chromosome:GRCh38:3:137269248:137269865:-1

137269865 TGATCAGCGGCTTCTGTGTGCCTTTTTTTATGCAGGAAATAATGGGTGAGCTGGGTACTC 137269806

137269805 CTGTTATCCCGCTCTCACAAATAATTAAGACTACAAAGGGTACAAATCAGTTTGAATGTG 137269746

137269745 GTGCACAAAGGCACAGGCTCAGCAAAGGCCTTTCTCTGTTTCTGTTTCTCCATCCCCCAC 137269686

137269685 CCCCTATTTTCTCCCTTTTTCATTTGTTCCTTTCTTTTGACAAGAGGTGGTTTCCAAAGA 137269626

137269625 CACCTGGGCAGGCAAGGCACTGGCCCATCCAAAATATTAATTCCCTGCCTCCCTC**CACCC** 137269566

137269565 **CCATCACCACCACCAGGG**CCTGCAGAATGATGTGAGTTTTCATCTCAGAGGATTTGGATT 137269506

137269505 CCTCTCAAGACTGGTTTTCACAGCACTAACTGCAGTGGAGGAAAAGAGAGAGAGAACTTC 137269446

137269445 CTGAATGGTGTCTCTCCCATATTTGCCACCCTCTTTGTTTGTTCATCTTCAAGTAAGAAC 137269386

137269385 CTGCTAGGTGTGGTGCTACCATGATCAGGGAGATGAAGAAAGGATCTCCTCCAGCCCTAG 137269326

137269325 ACCTTACTCCAGCAACCTCAGGAGAGTTTGGCACGCAGCAGGCCAGGCCAGCCAGGGACC 137269266

137269265 AGGGAGCTGGGCGCAGGA

C9-OFF6

>chromosome:GRCh38:19:18086722:18087339:-1

18087339 TACTCGGGAGGCTGAGGCAGGAGAATTGCTTGAACCCGGGAGGCAGAAGTTGCAGTGAGT 18087280

18087279 GGAGATCATGCCACCACACTCCAGCCCGGGCGACAGAGCAGGGCTCCATCTCAAAAAAAA 18087220

18087219 AAAAAAAAATTGGCTAGAGAGGGCCAGAGACCTGGCCAAGTGCATGGAACCACAGGTGGC 18087160

18087159 CCATGGGCCCATGGGGCTGGCATCTGGGTGTTCAGTTCAGGTCTGGGGCCAGCAGGGCAG 18087100

18087099 AACCCCCTAGAACCCTGACTTGCTCCAAAGTCAGACTTGCTCCAGAGTCTGTGGC**CGCCA** 18087040

18087039 **CCATCACCACCACCACGG**AGCTGACTTCCTGGGTTTCTCTTTCACTTTGACTTGCCTTAG 18086980

18086979 GGATGGGCTGTGACACTTTACTTTTTTTCTTTTTTCTTTTTTTTCAGTCTTTTCTCCTTG 18086920

18086919 CTCAGCTTCAATGTGTTCCGGAGTGGGGACGGGGTGGCTGAACCTCGCAGGTGGCAGAGA 18086860

18086859 GGCTCCCCTGGGGCTGTGGGGCTCTACGTGGATCCGATGGAGCCGCTGGTGACCTGGGTG 18086800

18086799 GTCCCCCTCCTCTTCCTCTTCCTGCTGTCCAGGCAGGGCGGTGAGTCCCCTGACCCTGGC 18086740

18086739 ATGGCGGCTCCTCTTGCT

C9-OFF7

>chromosome:GRCh38:7:97005950:97006567:1

97005950 AGGCTTTCTCCAGCCCCCAAAGTTTTTGATGATGACCATGACTACGATGGCTGACGGCTT 97006009

97006010 GGAAGGCCAGGACTCGTCCAAATCCGCCTTCATGGAGTTCGGGCAGCAGCAGCAGCAGCA 97006069

97006070 GCAGCAACAGCAGCAGCAGCAGCAGCAGCAACAGCAACAGCCGCCGCCGCCGCCGCCGCC 97006129

97006130 GCCGCCGCAGCCGCACTCGCAGCAGAGCTCCCCGGCCATGGCAGGCGCGCACTACCCTCT 97006189

97006190 GCACTGCCTGCACTCGGCGGCGGCGGCGGCAGCGGCCGGCTCGCACCACCACCAC**CACCA** 97006249

97006250 **CCAGCACCACCACCACGG**CTCGCCCTACGCGTCGGGCGGAGGGAACTCCTACAACCACCG 97006309

97006310 CTCGCTCGCCGCCTACCCCTACATGAGCCACTCGCAGCACAGCCCTTACCTCCAGTCCTA 97006369

97006370 CCACAACAGCAGCGCAGCCGCCCAGACGCGAGGGGACGACACAGGTGAGAGGCCGCTGGG 97006429

97006430 GCAGCTCGCTTCTCCCGCCTCCCGACTGCCCCCTACCCCGCCCGCCCGCTCACTTCCTCG 97006489

97006490 ACGCCCGGGCCTCCGCCGGCCCCCTCCCCCAGGCGGCCCCGCGCGCCCTTGGCCGGGCCC 97006549

97006550 CGTGCGCGCCCGCTCGCC

C9-OFF8

>chromosome:GRCh38:2:81033101:81033718:1

81033101 ACTGTAACAACTGTCTAGCTATTGTCTATGTTTTCTCAAGGCCTTAGGGCTGTAAAATCA 81033160

81033161 GAAGGTGGTGAAGCCAGACAGGCTTGTGTACTTCCCTTCAGGGTGTCAATCTCCCCTAGA 81033220

81033221 CCCCAGGTAGATCCAGAGATGCTGTCTGGGAGCCAGAACCTGGAGTCAGAAACCATAGAA 81033280

81033281 ATCCACCCAGTATTTTATTCTACTGCAGCTAAGCTGGCACTAAAACCATAAGATTCCATC 81033340

81033341 CTTTCCACTCTTTGTTCCCCCTTTCTAGAGCAGAAGAGTCTCTCCCCATGTCCTC**CACCA** 81033400

81033401 **CCACCACCACCACCATGG**GTAACACTATCAGTATTACCCATCGAAATACGGCCAGAGTAC 81033460

81033461 CACTGATGTTCACTTAAGGTGAAAAGGGCTTGCAGCTATTGCTGCCAGGTCTGGAACTCA 81033520

81033521 CTTCTTTGGGTGTTGTGCTCCCCTCTGGCCATGGGTAAATCCAGAAATGCCACCCAACAG 81033580

81033581 CCAATATCTGGAATCAAGGACCCCAAGTGCCTGCTTGGTTTTCTTTCCCCATTGTGGCCA 81033640

81033641 AGCTGCTACCTAAGCTGCGAAACGAAGTACCCTTTATTCTTACCTCTCCATTTCTCAACC 81033700

81033701 AGAAGGAGTCTATTCTCA

C9-OFF9

>chromosome:GRCh38:7:102295181:102295798:-1

102295798 CCTCCTTTCTTCCCTAACTCCTACTCACACTTCAATAATTAATCCAGAAGCCACTTCTTC 102295739

102295738 CAGGAAGCCTCCCGACACCCCTCCTTTCTCCCTGATCCAGGCCAGCACAAGTGTCTCCTC 102295679

102295678 TGAGCTCCCACAGTCTCCTCCTGCCTGTCTCTCTTGCATGGTGCCAGTTATAGGGTACTT 102295619

102295618 CAAGTGTCTGTCCTCATGGCTGTCTCCCCATCTGACACAGAGAGTGCGCAGGGCAAAAGT 102295559

102295558 CCAGGCTCCTGGAACTTGCTTCTTCCCTGAGGGGTGGAGACGGGAACACACCTGC**CACCA** 102295499

102295498 **CCACCACCACCACCATGG**CCTCCAGGGAAGGTCACGAGCCCAGGCCCAGGCCATGACCTG 102295439

102295438 CAGGGGTGGTATTTCCTGGGGTGGGGGTGGGGCCACAGCTGAACCCATGAAACCCTGTCC 102295379

102295378 CTGAGCTCCAAGCCCATCCATTTATCCAGTAGGCTAGTGCTCCTGACGGTGGCCACCTCT 102295319

102295318 CTCTCTATACCATTAGGACCCCCAGGGGGCTCAGAGCCAGTGACTTGGCAGCCTGGGAAG 102295259

102295258 GGTAGGTGTGGGGTCCCTGAGTGAGATGATTATGCCCCCACATGGGGCAGGCAGCGGCAG 102295199

102295198 GCAGGAAGTCAGCCCCAG

C9-OFF10

>chromosome:GRCh38:8:143429481:143430098:-1

143430098 CGTCGGGGGCACCTCGGGGAAGCCGGCGCTGGAGGATCTGTACTGGATGAGCGGCTACCA 143430039

143430038 GCATCACCTCAACCCCGAGGCGCTCAACCTGACGCCCGAGGACGCGGTGGAGGCGCTCAT 143429979

143429978 CGGCAGCGGCCACCACGGCGCGCACCACGGCGCGCACCACCCGGCGGCCGCCGCAGCCTA 143429919

143429918 CGAGGCTTTCCGCGGCCCGGGCTTCGCGGGCGGCGGCGGAGCGGACGACATGGGCGCCGG 143429859

143429858 CCACCACCACGGCGCGCACCACGCCGCCCACCATCACCACGCCGCCCACCACCAC**CACCA** 143429799

143429798 **CCACCACCACCACCATGG**CGGCGCGGGACACGGCGGTGGCGCGGGCCACCACGTGCGCCT 143429739

143429738 GGAGGAGCGCTTCTCCGACGACCAGCTGGTGTCCATGTCGGTGCGCGAGCTGAACCGGCA 143429679

143429678 GCTCCGCGGCTTCAGCAAGGAGGAGGTCATCCGGCTCAAGCAGAAGCGGCGCACGCTCAA 143429619

143429618 GAACCGCGGCTACGCGCAGTCCTGCCGCTTCAAGCGGGTGCAGCAGCGGCACATTCTGGA 143429559

143429558 GAGCGAGAAGTGCCAACTCCAGAGCCAGGTGGAGCAGCTGAAGCTGGAGGTGGGGCGCCT 143429499

143429498 GGCCAAAGAGCGGGACCT

C9-OFF11

>chromosome:GRCh38:X:82185541:82186158:1

82185541 CTTCATGTCACTGAGCTTCTGTAATACCAACATTTTAAATTCTTTTTTCTCAGATTTTGT 82185600

82185601 AAATGTAAATGTATTTGTGTTTAGAATCTTTTTTTATTTTTTTGAGAAGGAATCTCTCAT 82185660

82185661 GTTGCCCAGGCTGAAGTGCAATGACGAGATCTCGGCTTACTGCAACCTGCGCTTCCTGGG 82185720

82185721 TTCAAGTGATTTTCCTGCCTTAGCTTACTGAGTAGCTGGGAATTACAGGTGCCCACCACC 82185780

82185781 ACATACGGCTATACACACACAGTTGTGTGCACATGTGTGCACATACACCCACACA**CACCA** 82185840

82185841 **CCACCACCACCACCATGG**AATATTATGCAGCTATAAAAAATTAAAAAAACAAAAATGTGT 82185900

82185901 TTTGTAGCACCATGGATGAAATGAGGCCATTAACTTAAGTGTGTGTGATGTTTCCCTCCC 82185960

82185961 TGTGTCCATGTGTTCTCATTGTTCAACTCCCACTTATGAGTGAGAACATGCAGTGTTTGG 82186020

82186021 TTTTCTATTCCTGTGTTAGTTTGCTGAGAATGATGGTTTCCAGTTTCATCCATGTCCCTG 82186080

82186081 CAAAGGACATGAACTCATTATTTGGTATGGCTGCATAGTATTCTGTGGTGTATATGTGCC 82186140

82186141 ACATTTTCTTTATCCAGT

Tale-OFF1

>chromosome:GRCh38:14:67184155:67184771:-1

67184771 GGTTCCCAAAGTTGTATCCAGTCAGTAACTCTTCTGTGAACCCCTAACCTGTACTTTCAA 67184712

67184711 ATGCCAATGGGACATGTCTATCTTAGCACTCCTGGCATCTCAAACACATGCCCAAACTCA 67184652

67184651 ACTCCTCCATAAAACTCATTTTTTTCCTTCTTTTTATTAACGTTTAACATTTTCTCAGTC 67184592

67184591 AGCCAAGAGGGGAGACTTTGAATCATCTCTCCCTTCTATAACTTTCTTTCCAAAACTTGA 67184532

67184531 AATCAGACATAAAATTCTTTCCATTCTTTTCTCTCCAGAGACGTATCCATCTGTTCGGAC 67184472

67184471 **GTTCCCATCACCATCAC**CATCACCTCTACCCCCTCACCTGCACAATTGCTATAACTTAAC 67184412

67184411 TGGTGTCTCTCACTTGTTTCTTTCCATTCTAGTTTATCTGCCTCAAGGCTGCCAAAGCGT 67184352

67184351 TTTCCCTAAAAGGATATCTCTGATCATGATACGTCCCTGATCAAGAATCTCTAATGGTTT 67184292

67184291 TATTGTCTCTAGAATAAATCTAGACTCTTAATTTGGTATTTAAGAACTTTAATACCAAAT 67184232

67184231 TAACTTTAATAACTTTAATAACTTTTGGGCCTTAAATCAGTACTTTTTCAGTCTTACAAA 67184172

67184171 CATCTTATACCTATTTT

Tale-OFF2

>chromosome:GRCh38:12:68564372:68564988:1

68564372 TTCTTTCTTTCTTTCTTTCTGTTTCAATTTCTCTGGCCTAATTGTTGGGTGAGACTCTCC 68564431

68564432 TTGAATCTTACTTCACTCTTCTAAAAAACACAATTATTTCTAGGGAAAAATCATTTAAGT 68564491

68564492 TCTCACATTTTTCCTTTGTATTTATTTACAAAGCCTCTCAGGTATCAAGAAAGAGTTACT 68564551

68564552 TTTTATTTTTCTCTTTTTTTTTTTTTTTAGCCTCTGATCTTATTAAAAGGGAAGAAATAT 68564611

68564612 CTAAATAGTATTACAACAAATTACATAAATGTTAGCTTCAGCAAAGTAATGTTTTTAACT 68564671

68564672 **GTTCCCATCACCATCAC**CACCATCACTGAAAGCTGGTGAGAAAATCTACTGAAAGGGGTG 68564731

68564732 AGATTTCAGTAGATCTCACACACACACTACAATATTACATCTGACCCCTTTCAGTAGGTT 68564791

68564792 TTCTCACAACACAATGTAATATGCACACAATACAATGTAATATTCAAAAGATAATTGCAG 68564851

68564852 GATTTATCCTAAAATATTAATTCATGGTGATATAATTCAGTAATAAAAATGCACATTTCA 68564911

68564912 AGTCACAGAATGCATCCTATCACTGAAACTCTATGTATGACCGTATTTGGGGATGGCACA 68564971

68564972 TGTAAATCCAAATTTTA

1. DYRK1A#2

| TaC9 target locus | On target sites | gRNA off target sites | Tale off target sites |
| --- | --- | --- | --- |
| DYRK1A#2 | 1 | 5 | 2 |

On target：CAGGGGCATGCACCCCCTCTATA**CACACACCCCAGGAGTGCTGTGG**

>chromosome:GRCh38:21:37477144:37477761:-1

37477761 TAGGATTTCAGGACCCAGCTGGCACCAAAAAAGGGGGCTGGCAGCAGAGCACCATTCCCT 37477702

37477701 CCAACTGCTCCTAATAAGAAGAAATTCAGCTCCTAATGTCTCCAGACCCTGCTGCCCTCT 37477642

37477641 GACAAATATCTGCCCCTAACCAAGGCTTGCCCTCACAGTCCCCTTCCCTGATCCCAACTC 37477582

37477581 ATGGCTCTCGACACCATCTGACAGGCAATGAGGGGTTCCACCGTGAGCATCCTGCTAGAC 37477522

37477521 CGTTTCCACAGCAATTACAACAGATGATAGAGCAGGGGCATGCACCCCCTCTATA**CACAC** 37477462

37477461 **ACCCCAGGAGTGCTGTGG**CTAGCATCAATGATCCTGGATTGCTGTCCAAGTGCCACTGCT 37477402

37477401 CCAGCAAGTGCCATGCCTCAGTCAAAGGATTCCCAATGGGGGTTCTTGCAATCAACAGAT 37477342

37477341 CCTCAAAACCAATTATTTCCATCAAGGTGGTCACAGCCATTCCCCCTGGCCCATCTTTGT 37477282

37477281 GTTCCTGCCCAACTCTTCTGGACATTTGTGCTGGGAAATGAGCCAGAAGACCCTGACACA 37477222

37477221 ACATCTCTCCAAGGACACTCGGGTCAATCGGAGGGGCAGAGTGACAGTAAACACATCATA 37477162

37477161 CTAAGACCTCGCAAAAAT

C9-OFF1

>chromosome:GRCh38:7:138949441:138950058:1

138949441 TAAAAAATCAGTATTTTTTTAAAGTAAATGGAAGTTCTGATGTTTTCTTCCCATGCCCAA 138949500

138949501 CTGTGGAGACCGAAAGCCTATTCTGCTGTCTCTCGGCATACAGCTAATGAAGAGATGGGG 138949560

138949561 TTCCAATGCCAGCCTGTAGGACTCCACAAACACCTAAACCAGTATTCAAGCGCCAACCAC 138949620

138949621 CTGAGTGAACAAAGGTCCAATCTGGCTCAAGCTTAACAGGAGAGTACCAGGAAATGTTTG 138949680

138949681 CCTCAAGGTTAAAAAATTCCTAAAGTCACCAAACAGTAGTGTGGCTTCACATGAC**TGCAG** 138949740

138949741 **ACCCCAGGAGTGCTGAGG**ACCTATGACAGAACCCAGGACTGGCTATGACACGCCAGACTC 138949800

138949801 TCAGAGACCCACAGGTGGGTCACACATTCTAATACATCTGAGCCAACCCCAGCAGGGGCC 138949860

138949861 GGGGCAATTCACGCAATATCACGAGAGCAGCGAGTACACCTGACTTCAAGTCGGTGCCAA 138949920

138949921 GCCCCACAATCTTCCTAGTCGGTGCCAAATTTTCAAGAAGATGGCTGAGGCCACCTTATT 138949980

138949981 TCAGATCTGCACAGGAACAAGTTATCCCAGCACCATGAGCTAACACTTCGTGGTCTCCAT 138950040

138950041 TGTGTTAAAACGTGTGTA

C9-OFF2

>chromosome:GRCh38:10:14843201:14843818:-1

14843818 ACTGCTGTTGCCAAACGTCTGACTGCGATGACAGCATCCCTGCGGAGCTCATTGGCAGTA 14843759

14843758 TCTTGCTGAGCACTGGCCTCCTCACCAATAGCAATCAGCCTGTCCAGTTTTTCTTCCTCC 14843699

14843698 CGCTTTGAGAGTTCTCGTGCCAATCGCCTGTTTTCTGCCAACTCGGCTGCGATAGTCCTG 14843639

14843638 GCCACTGACCGCCTTCTTCGCCTTGCCCACCTTGGAGGGGGCTCACTGGTCAAGGGCCTA 14843579

14843578 TCCCCACCAGAAACAAAACCAGCTCTCGAAGAGGAGACTGGAGTCTGCGGTGTGG**AGGCT** 14843519

14843518 **ACCCCAGGAGTGCTGCGG**TTGGTGCTGGTGCAGGGGCTGGGCTCCCCGGACACACCCAGT 14843459

14843458 CTGGCCATGGGGCTGCTCTGACAGGGAGCAACTGCACCTCTGAAAAGGCTGTGGGAGCTG 14843399

14843398 CTGTAGCTGGGGGTCCCTTGAGAGCACCCTGAAAAACAAAGCAACAATGTTGCAAAGAGA 14843339

14843338 ACATTCCTGCTAAGGGAATGCTTGGAGCCAGCAGCAGAGCTGAGACAAATTATAAAACTA 14843279

14843278 TGGTTGTTCCTGGAAAGCCTCTTTCCACCAGGGACTGGGAAGAATCCATTTCCTTAATCT 14843219

14843218 GGACAATTGTGACCTGTT

C9-OFF3

>chromosome:GRCh38:20:16785433:16786050:-1

16786050 AAATATTCCTGACTTCATGGGTATTATATTTTATAAAGGAAGAAGAAAATTACCAATAAA 16785991

16785990 CATAATAAATAGATAAATATTTGCTACCCTCTGCCTGCACACACATGCAGGACTCACCTG 16785931

16785930 CAGGGATTCCTCCTGCCTCACTATCACCCACTGATGTGATATTGCTGTCACCCCTTATCA 16785871

16785870 GAGTGTTGTTGTCAACAAACTGGAAACACCTTAACCCCTCCAGCACATAGGGTGCTTAAC 16785811

16785810 CTCAAGAAGCCAGAGAAAAAACCAAGTGTCTGGTCTCAGCCACCCAGTGTTAGCA**CACAC** 16785751

16785750 **AGCCCAGGAGTGCTGTGG**CCCCCTAAAGTCATCCAGAAATGAAGCCAATCAACTAAGTCC 16785691

16785690 AACTTATATATCAGTTAAACCCTAAAGGGTATCAAAGAATGTAAGAGCAAAAAGCCATAT 16785631

16785630 CCAAAGGATAGCAACTTCAAACATTAAAGGAACATCAGCCCACACGATTGATAAAGAACC 16785571

16785570 AGCACAAGAACTCTGGCAACTCTGAAACCCAGAGTGACTTCTGACCTTCAAATGACTGCA 16785511

16785510 CTAGCTCCCCAGCAATGGTTCTGAGCCAGACTAAAATGGTTGCAATGACACACATAGAAT 16785451

16785450 TCAGAATCTGGATAGCAA

C9-OFF4

>chromosome:GRCh38:16:88059648:88060265:1

88059648 CCACAGATGAAGCTTTTTGCAGGAAGATGTGAGTTTGGTGGGGTTCTGGGTGCTTTGGGG 88059707

88059708 CTTCCCGACGGAGACGTGTGCTACTCCTCGCGTCCTGTCAGGGCCCTACCAGCTGCACTG 88059767

88059768 TCGGACGCGACACATGCCATGCCCCCTCCTCTGCTCCCTGTGTGAGACCGCATGGTCATG 88059827

88059828 GACATCCCGCTGAGTGCGCCTAGGTCAGATGCACTTGAGACCCGGCATTGGGATGGAGTC 88059887

88059888 ACCTGGAGCCACGGCCTGGTGGGGAAGGGTCACATTTAGGCCCCAGACCTGCACA**CACAC** 88059947

88059948 **ACCCCAGGAGGGCTGAGG**TTGGCACAGGTGGGGTTCTCCGTGTAGTGTGTGGTGAGACTG 88060007

88060008 GCTTGGCGCAGCTGTCTGTCTCTGGGCCTTCCTCTGAAGATGGCAGGTGCTCTGACTTAC 88060067

88060068 CCAAGTTGGGGGTGCTCTGCTGTGGCCGAAGGTGCCGAAGTGTGAGCGGCTTGTGTGAGA 88060127

88060128 AGTGTGCAAAGGTGCTCTTAGCCACCAGCACATAGCAACCACCTGTCGGCACGAGTGGCA 88060187

88060188 AGGCCGTATGTTTCTAAGCATAACGTAGTTTGAGGTGTGTGCAGAGACCGCCGTGCGTTG 88060247

88060248 CCCTCCGGGCAGAGCCCA

C9-OFF5

>chromosome:GRCh38:X:140865565:140866182:1

140865565 GATCCTGCACAGACACTGGCAACCCTGCCACCACCCTACCCCTGCTGCTGGCCTACACCT 140865624

140865625 TCCTTCACGTTGGCACTCTGCTGTGCTGTTGTGGCTGCTGGCATGTGCAAGTGAGCATGT 140865684

140865685 TTACCACTGCCACTGCCACTGCCACTGCCCCTGTGAAGCACTTTGGCTGGCACACCCCAT 140865744

140865745 GAGTGTTGGGGCCAGTGGTCTGCAAACACCCTGGCCCCTCCAGTGCATCAGGTTCCTAAC 140865804

140865805 CTCAAGACATCAGAGATCAAGTCCACGGGTCCAATACCAGTCCCCAGAGTTATAG**CACAC** 140865864

140865865 **AGCCCAGGAGTGCTGAGG**TGAGCTTCGGCACCCTGAAATCTTCCAGTATTGGAGCCAGTT 140865924

140865925 GACAGAACCCACCTTATACCACAATCATACCACCAAAGGCATAAAAGAAGATAAAAACAA 140865984

140865985 CAACAACAAAAAATTCCACACAAAGGACAGCAGCTTCAAGGATTAAAGGAACATCAGTGC 140866044

140866045 ACATAGATGAGAAAGAATGAGCACAAGAACTCTGGCACCTCAAAAAGCCAGAAATGTCTC 140866104

140866105 CTTACATCTAAACAACCACACTAGTTCCCCTGCAATGGTTCTTAACCAGGCAGACACGGC 140866164

140866165 TGAAATGACAGACCTTGA

TALE-OFF1

>chromosome:GRCh38:X:71148484:71149100:-1

71149100 GCTCTGAGCCTGCCTTAGGCCACCAAATCCTCCCTGCAAGGCAGCCAGGGCCCTCAGATG 71149041

71149040 AGGTGGCGCTTATGCCTGCTTACTGCCCCCTGAGCCTGGGCTACTTGGAGGAAGGCTGGG 71148981

71148980 TCCTGGGGGCAAGGGCAGGTGGGCAGGCAGCCCTCCCTCTCTCTCTCTCTCCCTCCCCGC 71148921

71148920 CGGCTCGCTACCTACCTCCTTTCCTACAAATTTTCTTGTTGGGCTTTCGGGCGCATTCCT 71148861

71148860 TGGAAATCCGCTTTACTGTAAAATGAATAAAAAACTAAAAAATAACAGGAGGCCTCTGCC 71148801

71148800 **CAGGGGCATGCACCCCC**CACCCCCCAGGCCCCAAGAGCTGAGCTGCTCTGCAGGCGGCAC 71148741

71148740 ATGGCAGAGAGCCTGTCCCACCATCTCCTTGTTTCTGTCACTTACCTTAGTGTGACCCCC 71148681

71148680 GCGGGAGGAGGCATCAGCTAGTGAAAGAGGAGGCACCTAAGCATCACCCACCAGGCTTGC 71148621

71148620 TGCCCCACCCCCCACACACCCAGTGCCCTTGGCCACACACACCTACCACCTTTCGAAGCA 71148561

71148560 GGTGGAATGGCAACAGGGGGGCCTGGTGGAGCCAGCATCCTCCCGCGGGTAGCTGCCAGC 71148501

71148500 CGTGACCCATACTCCCC

TALE-OFF2

>chromosome:GRCh38:8:82370709:82371325:1

82370709 GGGAAGTCTGCACCTTCTGCAATATGAAGAGTAGTGTCATTTCCTCCAACGCCAATATTA 82370768

82370769 CAAACAATATCACAGGAAGGTGTACACCCTCTCTGATATGAGGTGTAATATCATCTTCTC 82370828

82370829 CATACCGGAATATTACGAACAATATCGCAGAGGTGTGTATACCTTCTGCGATGTGGGGAG 82370888

82370889 TAATATCATCTTCTCTTCCCCTGGATATTATGAACAATATCACAGACGTTGTAAACAGAC 82370948

82370949 GGTGTCTATGATATCGAAAAAAATATAATCTCCCCCCCCCCAATATTATGAACATTATCA 82371008

82371009 **CAGGGGCATGCACCCCC**TCTGATATTGAGTAATATCATTTTCTTCTTTCCTGAATATTAA 82371068

82371069 AAACAATATCACAGGGGAATGTACACCCCCTGTGATATTGGGAGTAATATCATCCTCTTC 82371128

82371129 CCCACTGGATATTAAAAACAATATCATAAAGGATGTACACCCCCTGTGATATTGGGAGTA 82371188

82371189 ATATCATCCTCTTTCTTCCTGGATATTACAAACAATATCACAGGGGGGTATACACCCCCT 82371248

82371249 GCGATATTGAGAGTAATATCATCCTCACCCTACTGGGATATTATGAAAAATATCACAGGG 82371308

82371309 GGATGTACACCTTCTGC

1. POU5F1(OCT4)

| TaC9 target locus | On target sites | gRNA off target sites | Tale off target sites |
| --- | --- | --- | --- |
| POU5F1(OCT4) | 2 | 5 | 1 |

On target：**GTGTCCCAGGCTTCTTT**ATTTAA**GAAAAAAGTGATACATGATGTGG**

>chromosome:GRCh38:6:31164078:31164700:1

31164078 TGGGAAAGCAAAAAGCCAGCTCTGAACAGGTAACAGCTACATGGTGACTGAGTCTATGGG 31164137

31164138 CAAAAGTTCTTGCATCACAGGCTTTTGGGAACTAGCCTATCACAGGGCCCTGTACAAATA 31164197

31164198 AACTTGGCTGCAATCCCAGCTCTCCCTCTGATGTTGTGTGACCTTAAGGAGTGTAAATGG 31164257

31164258 CACCTTAGTTTCAGGGTCACTTGGGTATGAGCATTGGATATTCCCATCCCTACCTCAGTA 31164317

31164318 ACTGAAGGACAAACCAAGATAAGTGTGTCTATCTACT**GTGTCCCAGGCTTCTTT**ATTTAA 31164377

31164378 **GAAAAAAGTGATACATGATGTGG**GATTAAAATCAAGAGCATCATTGAACTTCACCTTCCC 31164437

31164438 TCCAACCAGTTGCCCCAAACTCCCCTGCCCCCACCCTTTGTGTTCCCAATTCCTTCCTTA 31164497

31164498 GTGAATGAAGAACTTAATCCCAAAAACCCTGGCACAAACTCCAGGTTTTCTTTCCCTAGC 31164557

31164558 TCCTCCCCTCCCCCTGTCCCCCATTCCTAGAAGGGCAGGCACCTCAGTTTGAATGCATGG 31164617

31164618 GAGAGCCCAGAGTGGTGACGGAGACAGGGGGAAAGGCTTCCCCCTCAGGGAAAGGGACCG 31164677

31164678 AGGAGTACAGTGCAGTGAAGTGA

ON target 2

>chromosome:GRCh38:8:127416846:127417468:-1

127417468 GAATGGTTAATGCTCACATTTTTGTGGAATATGTGAGGGAATGGTGAGGGCCGAACTATG 127417409

127417408 CCGTTAGCGGTCAAAAGAGGTTTTGTATCAGGACAAATAGGATGTCTTGAAGAAAAACTA 127417349

127417348 CAGCTACCATCAAGTAGCACTCTAGGAAAAGGTAAAAAGGAGGAAAAAAGCAAGTATTTA 127417289

127417288 TTGAATACCTACTACGTGCCACACATTTTACCACACTTAAAAAATTATTACTCAATTATG 127417229

127417228 TTACTTTTCTTTTCTTTCTTTTTTTTTTTTTTTTACT**GTGTCCCAGGCTTCTTT**ATTTAA 127417169

127417168 **GAAAAAAGTGATACATGATGTGG**GATTAAAATCAAGAGCATCATTGAACTTCACCTTCCC 127417109

127417108 TCCAACCAGTTGCCCCAAACTCCCCTGCCCCCACCCTTAGTGTTCCCAATTCCTTCCTTA 127417049

127417048 GTGAATGAAGAACTTAATCCCAAAAGCCCTGCCACAAACTCCAGGTTCTCTTTCCCTAGC 127416989

127416988 TCCTCCCCTCCCCTGTTCCCCATTCCTAGAAGGGCAGGCACCTCAGTTTGAATGCATGGG 127416929

127416928 AGAGCCCAGAGTGATGACGGAGACTGGGGGAAAGACTTCCCCCTCAGGGAAAGGGACTGA 127416869

127416868 GGAGTACAGTGCAGTGAAGTGAG

OFF1

>chromosome:GRCh38:12:8133248:8133870:1

8133248 GCCATGTTGGCCAGGCTGGTCTAGAACTCCCGACCACAGGTGATCCGCCCGCCTCAGCCT 8133307

8133308 CCTAAAGTGCTGGGTTTACAGGCGTGAGCCACTGTGCCCAGCCTTTTCTGTCTAGTTTGT 8133367

8133368 TTCTGTTGCTCAGTTGACATTTTCTGTTGTTTTGTCTTCCAATTCACTAATCTCTCTATC 8133427

8133428 CCCTTCATTCTGCTGAGTCCATCCACTGAGCTTTTTATTTTGGTTATTATATTATTCAAT 8133487

8133488 TATAAAATTTCCATTTTTTTTTTTTTTGTCTATCTACTGTGTTGAGGCTTCTTTATTTAA 8133547

8133548 **GAAAAAAGTGATACATGATGTGG**GATTAAAATCAAGAACATCATTGAACTTCACCTTCCC 8133607

8133608 TCCAACCAGTTGCCCCAAACTCCCCTGCCCCCACCCTTTGTGTTCCCAATTCCTTCCTTA 8133667

8133668 GTGAATGAAGAACTTAATCCCAAAAGCCCTGGCACAAACTCCAGGTGCTCTTTCCCTAGC 8133727

8133728 TCCTCCCCTCCCCCTGTCCCCCATTCCTAGAAGGGCAGGCACCTCAGTTTGAATGCATGG 8133787

8133788 GAGAGCCCAGAGTGGTGACGGAGACAGGGGGAAAGGCTTCCCCCTCAGGGAAAGGGACCG 8133847

8133848 AGGAGTACAGTGCAGTGAAGTGA

OFF2

>chromosome:GRCh38:1:155434163:155434785:-1

155434785 TCTGCCTCCCAGGTTCAAGTGATTCTCCTCCTGAGTAGCTGGGATTACAGGCGCACACCA 155434726

155434725 CCACACCACCATGCCTGGCTAATTTTTTTGTATTTTTAGTAGAGACGGGTTTTTACCATA 155434666

155434665 TTGGTCAGGCTGGTCTCAAACTCCTGACCTGATGATCCTCCCGCCTCGGCCTCCCGAAGT 155434606

155434605 GCTGGGATTACAGGTGTGAGCTACGGCGCCCCACCATCCTAAATTCTTATATACTGTTAG 155434546

155434545 ATCTTAGGGTCTTTTCAATTTTTTTTTTTTTTTTTTTTTTTTACTGTGTCCCAGGCTTAA 155434486

155434485 **GAAAAAAGTGATACATGATGTGG**GATTAAAATCAAGAACATCATTGAACTTCACCTTCCC 155434426

155434425 TCCAACCAGTTGCCCCAAACTCCCCTGCCCCCACCCTTTGTGTTCCCAATTCCTTCCTTA 155434366

155434365 GTGAATGAAGAACTTAATTCCAAAAGCCCTGGCACAAACTCCAGGTTCTCTTTCCCTAGC 155434306

155434305 TCCTCCCCTCCCCCTGTCCCCCATTCCTAGAAGGGCAGGCACCTCAGTTTGAATGCATGG 155434246

155434245 GAGAGCCCAGGGTGGTGACGGAGACGGGGGAAAGGCTTCCCCCTCAGGGAAAGGGACCGA 155434186

155434185 GGAGTACAGTGCAGTGAAGTGAG

OFF3

>chromosome:GRCh38:10:68009680:68010302:1

68009680 CTCTTCTTTTCATAAAAGATTTCTCTATACCAGGAGATGCTGCATGATAGCATTTTACCC 68009739

68009740 ACGGAACTGCTTTCAAAAGTAGAGTCAAGCCTCTTAACCCCTGCTGCTGCTCTGTCAACT 68009799

68009800 AAGTTTATGGAATATTCTAAATACTTTGTTGTCATTTCTATAATGTTCATAGCATCTTCA 68009859

68009860 CCAGCAGTAGGTTCCATCTCAAGAAAACACTTTCTTTGCTCATCCATAAAATATAAATGG 68009919

68009920 ATGAGGAGTATTTTTCTTTTTTTTCTCTCTACCTACTGTGTCCCAGTCTTCTTCATTTGA 68009979

68009980 **GAAAAAAGTGATACATGATATGG**GGATTAAAATCAAGAGCATCACCGAACTTCACCTTCC 68010039

68010040 CTCCAACCAGTTGCCCCAAACTCCCCTGCCCCCACCCTTTGTGTTCCCAATTCCTTCCTT 68010099

68010100 ACTGAATGAAGAACTTAATCCCAAAAGCCCTGGCACAAACTCCGGGTTCTCTTTCCCTAG 68010159

68010160 CTTCCGCCCTCTCCCTGTATCCCATTCCTAGAAGGGCAGGCACCTCAGTTTGAATGCATG 68010219

68010220 GGAGAGCCCAGAGTGGCGACAGAAACAGGGGGAAAGGCACTAAGGAACACAGTGCAGTGC 68010279

68010280 AGTGCAATGAGGGCTCCCATAGC

OFF4

>chromosome:GRCh38:4:160472722:160473344:-1

160473344 TATTTATGTTATTTTTTTTTAATTTCCTAGATACAGGTAGATAGAATTTAAGTAACATTA 160473285

160473284 GGATAAGGACAATTTAAAAGTAAATGTGTAAAATGTGAATGTTGCCACACAGTGAACAAT 160473225

160473224 GGGAACAATGCACTTGGAAGATTTTTGTTAATTTTTCTGAATTTTTTTTCTTTACATTAA 160473165

160473164 TTTTCTTCCCTATATTGTAGGGGTCGGAAGTCTTAGGATTATGCTCTTCTGACTTTCTTC 160473105

160473104 TATCTAATGTTCTAGATGTAGCTTACAATTTATCAGTGATATATACATATAAAAGAATTA 160473045

160473044 **GAAGACAGTGATACATGATGTGG**ATTTTGACAGATGTGGAGGTGTTTTGTAGCTGTCTGT 160472985

160472984 CCTGCCAGCCATCTTCTTCTTGCCTGCACGTGGAGGTAGCTGCAATTTCCTGGGAAACTG 160472925

160472924 ACCATGGCTAGTCTCCAGCCCATAGCTGTGGCAGTTAATATGAAAGCCAAGGTGTTTTGT 160472865

160472864 TTTGTTTTTTTTTTTTTCCCCACTCCTCTAGCATTTTCAATGCTGTTGTGGGAAAATATG 160472805

160472804 TATAATGGTTTCTGTTTTACGAACTAGGCCATGACTGGCAAAGTATTTCTGAAATGATTT 160472745

160472744 TTATACTCCTTGAATGTATTTTC

OFF5

>chromosome:GRCh38:15:59929476:59930098:1

59929476 CATGATCTCGGCGCACTGCAACCTCTACCTCCTGGGTTCAAAAGATTCACCTGCCTCTGC 59929535

59929536 CTCCCAAGTAGCTGGGATTACAGGCACCCGCCACTATGCCCAGCTAATTTTTTTGTATTT 59929595

59929596 ATAGTAGAGATGGGGTTTCATCATGTTGGCCAGGCTGGTCTCGAACTCCTGACCTCAGCT 59929655

59929656 GATCCACCCACCTTGGCCTCCCAAAGTGTTGGGATTACAGGCGTGAGCCACTGTACCTGG 59929715

59929716 CCATCCTGTTGCTTTTATTTGCACAGAACATGAACCTTAATTCAAGTGGCTTAATTAAAA 59929775

59929776 **AAAAAAAGTGTTACATGATGAGG**AAATCCTGAGTTGGGCAGCTCCATCAGCAGCTGCTCA 59929835

59929836 GTGTAGTCACAAAGTGCACAGGTCCCTTCCATCTCTTCCCATTGCCTTCAAGTTGATTTA 59929895

59929896 GGATGCATTTCCGAAAGATGGACTAGGAGTGGGGTTATAGTTGATAAGTCATTACTTAAT 59929955

59929956 GTCATTGCATCCCGTTAGGAGGAAGAGGTATGTAGAGAAGGGTTTAGAAGAATCATAGAA 59930015

59930016 AATTTCAAAAACAAGAATCCTGGTCATTGTTGTCCATGCCTATGTCCATGAAGCCTTCTG 59930075

59930076 TGAAAAGGCTATCCTTACAGTGG

TALE-OFF1

>chromosome:GRCh38:4:808361:808977:-1

808977 CACCAATTTTTGGCATTGCTCTTATGTTAATTTCACAAAAATAACCCAGAGGATTTCCTT 808918

808917 CTTTTTCTCCGTTCCAAGAACATTCTAAATATCATTGGAATTATCTGTTTCTTGAATGTT 808858

808857 TGAAAGAATCCATCTGTGAATTCTTAGACATCGTTCTCAGATTTTTTACAACGGGTTTTA 808798

808797 AAGACAATCCATTTCATTCAAGTTTTTAAGTGTATTAGGAAAGTGTGTGTAAAATATTAT 808738

808737 TTGACCATCTTATTTCCTGTCTGGGTGTGGCTAGTTCTCAATCGTTTCCCCTCGTTTTGT 808678

808677 **GTGTCCCAGGCTTCTTT**CTGTCTTCTCACTCTGAGCAGCTGCCTGATGCCTGTTTTATGG 808618

808617 GAGGTTCAAGACCCACCTCCTGAACCGCTGACTCTTTTGACATTTGTCTTTCTGGACGGA 808558

808557 TTCGTTTTGCCGTTAGCTTTGCAAACTCCTTTCCTCTGCCTGCCTTGGTTTGTTTGGTTG 808498

808497 CTTTTTCTGTGGCAATTGGAGTGAGGTGTTTTTTGCTGTGTATGCCTGGGCTTGCCGGGA 808438

808437 GCGTGAGCGTTTCAGGGAATGAACAGCATGGGCCACCCCTGACATGCCGCTGGATTGAGG 808378

808377 CCTCGATACATCCAGTT

1. VEGFA

| TaC9 target locus | On target sites | gRNA off target sites | Tale off target sites |
| --- | --- | --- | --- |
| VEGFA | 1 | 4 | 0 |

On target：**AGCCGGAGGAGGGGGAG**GAGGAA**GAAGAGAAGGAAGAGGAGAGGGG**

>chromosome:GRCh38:6:43770706:43771323:1

43770706 GCTGACGGACAGACAGACAGACACCGCCCCCAGCCCCAGCTACCACCTCCTCCCCGGCCG 43770765

43770766 GCGGCGGACAGTGGACGCGGCGGCGAGCCGCGGGCAGGGGCCGGAGCCCGCGCCCGGAGG 43770825

43770826 CGGGGTGGAGGGGGTCGGGGCTCGCGGCGTCGCACTGAAACTTTTCGTCCAACTTCTGGG 43770885

43770886 CTGTTCTCGCTTCGGAGGAGCCGTGGTCCGCGCGGGGGAAGCCGAGCCGAGCGGAGCCGC 43770945

43770946 GAGAAGTGCTAGCTCGGGCCGGGAGGAGCCGC**AGCCGGAGGAGGGGGAG**GAGGAA**GAAGA** 43771005

43771006 **GAAGGAAGAGGAGAGGGG**GCCGCAGTGGCGACTCGGCGCTCGGAAGCCGGGCTCATGGAC 43771065

43771066 GGGTGAGGCGGCGGTGTGCGCAGACAGTGCTCCAGCCGCGCGCGCTCCCCAGGCCCTGGC 43771125

43771126 CCGGGCCTCGGGCCGGGGAGGAAGAGTAGCTCGCCGAGGCGCCGAGGAGAGCGGGCCGCC 43771185

43771186 CCACAGCCCGAGCCGGAGAGGGAGCGCGAGCCGCGCCGGCCCCGGTCGGGCCTCCGAAAC 43771245

43771246 CATGAACTTTCTGCTGTCTTGGGTGCATTGGAGCCTTGCCTTGCTGCTCTACCTCCACCA 43771305

43771306 TGCCAAGGTAAGCGGTCG

C9-OFF1

>chromosome:GRCh38:3:15511848:15512465:1

15511848 AAAGGGCAGTCCAGTTTCAACTCCTCATGATCATAACCCACCCGACAGTCCCCTCATACG 15511907

15511908 GGGGACAAGTCCAAACACTGGAACAAACCCGGAATCCTAGTGAAGTAAGAAGAGCAGGTG 15511967

15511968 GGCAGGAAAGTCTCTCCAAGTAGGATGGCCAATGTCGCCCCATTCCCTGTCTTCCCGGGT 15512027

15512028 CCCCAAGAAGCCCCATGCCAGGCCCGAAACCCCTGGAAAGTCCTCTGTGGAGCATGGTCC 15512087

15512088 CAGGACAGAACTCCCAGCAGCAGCAGGAAAGCTCCTGATGCCTTCTCCAAACTTC**TCAAA** 15512147

15512148 **GAAGGAAGAGGAGAGTGG**TACAGAAGGATGAGTGAGGAAAGTGTGGGATGCCTGGGAGGG 15512207

15512208 AGGAACCTGCCCGACTCACACCGATGCTTCCTTCTTTTCTTTCCTCTCTTTTTTCATTTG 15512267

15512268 CAGCCCGCAGCTGGTTGATGGTGCAAAGCCTGTAGCAGCCACGAGGCCTCTCCTTCAGCT 15512327

15512328 GTCACCAGCTCCCAAACACGCGCCCCATGCCTGTGTTTATGCTGGTTGCCATGAGAGATA 15512387

15512388 AGTGCGGGCTTGAATGGAGCTAAATGCTCCTGGGCTGGGAGATGGAAGAGGGCCAGGTGT 15512447

15512448 ATTTTTAAAGCATCTGGG

C9-OFF2

>chromosome:GRCh38:6:43803640:43804257:1

43803640 AGGCTTGGCCCCTTGTTATTTCCTGCTTTCCTGGGGCTGTGGCTGTGGCTGTGGGAGACT 43803699

43803700 TGACCCAAGAACGGACAGCCTCTCTTTTTCTGCTCTGCCATAGAGCAGAGCCCCAGCCTG 43803759

43803760 GAACACACGAGCCCTCTGAAATTACCTGCACAGTCTTGGGGGTATGTGAATGAATGTCCT 43803819

43803820 TTGGGGAAAGAAAGTCCTTATCTTTTATCAGATTCTCCAAGGGATTTCTGCCTTACAAAG 43803879

43803880 TTTAGAAACCTGTATTTAGAAGGCCAGGCTGAACTAAGAAGATAAGTCATAGGAG**GAGGA** 43803939

43803940 **GAAGGAAGAGGAGAGAGG**ACCCAAATCCTTTTATTTTAAAATGTCTTTTCTGGACCTTAG 43803999

43804000 TTTCCTCATCAGTAAAATTAGAAGTTTGGGTGATCTCTGGTAACATCCAGCATTTAGAAT 43804059

43804060 AATAATGCCTAATTTTACTTTGTACATTATCTCCTTTGCTCCTTTCAAAAGCCATGTGAC 43804119

43804120 TGTGTTTCTCATCAGCATCCCTGTTTTTCAGGTGACAACACTGAGGTCTGGTGACTCACC 43804179

43804180 CAGGGTCATGCAGCCTGTGAGTGGCAGAGTGAGCCGGGAACCTGGCTGCTGGCTGAGAAA 43804239

43804240 TTTCAGGGCTTCTTCTTT

C9-OFF3

>chromosome:GRCh38:16:47959941:47960558:1

47959941 TTCTATAAAAGATGAGGGACCTCTAATACATTTCTTAACCCCTTTGAGCCTAAGTTTCTT 47960000

47960001 CAGCTGTAAAGTAAGGATGCTAAAACCACAATTATCAAGGTGGTTTTGAGGATTCAATAG 47960060

47960061 ATAATACCAGTGGGTGCTGTGCACAGTGAGCAAAGGCTGGTTCTTGTTCCAAGCACTGAG 47960120

47960121 CATGGCTGCATGGGCTCTGAGAAGGCAGAAAGGAGAAAGCAGGGAGGAGATGGAGCCACA 47960180

47960181 GGAGAGCTTGGGTTGGGCCTTGGAATGTGGGAGGGTATGGGATGGGGAAGAGGAG**GAATG** 47960240

47960241 **GAAGGAAGAGGAGAGAGG**CAGGTAGGTTTCAGTGAGAAAAAGGCACAAAGGCAGGAAGAG 47960300

47960301 AGCTTGCCTTGAGCCAGAGGAGAAAAACATCAAAGGCCCAGTGAAGAGACCTGCAGAGGC 47960360

47960361 TTAGATTTCATGCCATGTGTGAGCCTTGGGTAGGGTTGTTAGCCTGGGAGGAACTTATTT 47960420

47960421 CTTACAACATATCTCTTTATTAAACATCAAGAGACAAATCAAACTGACCTTGTGGAGCTC 47960480

47960481 AACCTTGGGTTTGTAAAGTGTTTGTATAATGCAAAAAAAATAGTGAATCACTTGGGACCT 47960540

47960541 CTGCTGAATATTTTGGCA

C9-OFF4

>chromosome:GRCh38:1:44456845:44457462:-1

44457462 ATGCTTCAGTATCTCTGTGTCATTTTCTTTGCCTGGAATTATCTTCACTCCCTCTCTAAT 44457403

44457402 TGGAGAAATCTTACTCATAATTTTTTTCATTCATTTTTTCATTCATTCAACAAATAGTTC 44457343

44457342 TGGGCACCTATTGAGTGAAGAATGAACTAATATATTTCTATAATATATATAAAATGATGT 44457283

44457282 TTCCCTATTGAAATGTTATGATAAGCTATTCTTTTCCTTTGAAGCATTTATCTCAATTTA 44457223

44457222 CTATTTTATATTTATTTGTGTGTTCATTTGTTTAGTGTTCAAGGGAAGGGGCAGA**GAAGA** 44457163

44457162 **GGAGGAAGAGGAGAGAGG**AAGGTAGAGTGTTGGGAGGGTGTTGGGAGAGTATATATAGCT 44457103

44457102 TGCAGGGAAGGGGAAACGGTTGGGAAAACTCTAAGGAATTAACATTGGAGCTACAGCTGA 44457043

44457042 AGGATACGTGGGTGTAACTGGGCAAGACTGGCGGCAGTTGGGGATAGGTAGGAGCAGGAG 44456983

44456982 GGGTTGGGGGGACGTGTGTTCCAGATCCAGGGAACAGCGGGTGCTAAGACACTGAGGCAG 44456923

44456922 AAGGAAGGGTGACATGATGGAGAAACTGAAAGAAGTCCAGTGTGATGGGATTGCAGGATG 44456863

44456862 TCCTACCCCAGATGGAAG

1. AAVS1

| TaC9 target locus | On target sites | gRNA off target sites | Tale off target sites |
| --- | --- | --- | --- |
| AAVS1 | 1 | 4 | 6 |

Ontarget T**AAGAACC*GAGGACAAGTAGTGC***ACAGAA**GACAAAAGATCCCGCTCTCGTGG**

>chromosome:GRCh38:19:55118093:55118715:-1

55118715 GCCTTGGGGCCCTGCCTTGCACCCCACTGTGCTTTTAGGGGTGTGGCCCTGTGGGCTACA 55118656

55118655 TTTCCCAGCGTGCCCTAGGATCGTGGTCCTGACCTTTTCACCGCAAATTTCATTTCCCAG 55118596

55118595 CATGCCCTGCGAGGTCACCCAGGGAAGCTTGGAAGTGGACTGTCTTTCATGGCTTCTAGG 55118536

55118535 TTGCGGACTCAGTTTCCCATGGTGCCCTGGTGAACCCATTCATTCATTCATTCCAAAAAT 55118476

55118475 ATTTGCGATCAGGTTCTCTCAGGTTCTGTTCT**AAGAACC*GAGGACAAGTAGTGC***ACAGAA 55118416

55118415 **GACAAAAGATCCCGCTCTCGTGG**AGCTTATTTCTAGTTAAGGGGTCAGGAAAAAAACCAA 55118356

55118355 AATAAATAAAACCCAGCTGGGCATGGTGGCTTACGCTCGTCATCCCAGCAGGATTACAGG 55118296

55118295 CCGAGGCGGGCGGATCATGAGGTCAAGAGATCAAGACCATCATGGCCAACATGGTGAAAT 55118236

55118235 CCCGTCTCTACTAAAAATATAAAAATTAGCAGGGCGGGGTGGCGCGCGCCTGTTATCCCA 55118176

55118175 GCTACTCTGGGGACTGAGCCAGAATTGCTTGAACCTGGGAAGCGGAGGTTGCAGCGAGCC 55118116

55118115 GATATCGCGCCACTGAACTCCAG

C9-OFF1

>chromosome:GRCh38:X:118489420:118490042:1

118489420 ACTACACCTGGCTAAGTTTTGGCATTTTTAGTAGGGATAGGGTTTCACCATGTTGGCCAG 118489479

118489480 GCTTGTCTCGAACTCCTGACCTAAGGTGATCCGCCCACCTCGGCCTCCCAAAGTGCTGGG 118489539

118489540 ATTACAGGTGTGAGCCACCATGCCCAACAAAAATATACTTTTAAAATTAATTTCACCTGC 118489599

118489600 TTCTTTTTACTAATTTTTTAATGTGGCTACAAGTGAATTTATAATTACATATGTACTCAT 118489659

118489660 ATTATATTTCTGTTGGGCAGTGCTGCTTTAGGCACTGATGATAAAGCAGTGAACAAAGGA 118489719

118489720 **GACAAAATATCCCGCTTTCATGG**AACGTGCATTTTAGTGCGGAAGACAGATGATAAAATA 118489779

118489780 AATATATAAACATATATAGTATATCAAATGGTGACAAGTGCTTTGGAGAAAAACAAAGAA 118489839

118489840 AGCAAGTTAGATAGGAGATGGTAGGAGGGTTGAACTTTTAAATACGAAGGTCAAGGAACG 118489899

118489900 CCCAAAATCTAGATGTTGAGAGAGTAAGTAATGAAGCTTCTTCCGCTGGGTAGAAGCAAC 118489959

118489960 AGTAAGTGAGGAGGCCTTGATGATGGACAAAGCCTTGGCAATTCAGGGACCCTCAAGGAG 118490019

118490020 GCCTGTGTGGTAGATCATGGAGT

C9-OFF2

>chromosome:GRCh38:15:29899632:29900254:1

29899632 GCCACATCTACATCACTACAGGTTTGCTTGAAGTTTAAAGCTATTCTACATTAGGAGTTC 29899691

29899692 AATAATCATAATAAAGTTGTCAGTTTTCTTTTTACCTCCTAATCAATGTGTTAGTCCCTG 29899751

29899752 AAGAAAATCATTAAGTAAAAAGGACCCCACCGACCCACTCACCCACACACTAACACAAAA 29899811

29899812 TAGTTGTCCAATGGCAGGAAGTTGGATTAACTGATTAATTTATTCAGGAAATATTTATTT 29899871

29899872 ACATTTTGATCTACTCCATGCCAGATACTGTTATAGGTACTGAGGATACAGCAGTGAGCA 29899931

29899932 **GGCAAAAGCTCCTGCTCTCGTGG**AGCTTCTATTCCAGTAGGGAAAACACAACTAATAAAA 29899991

29899992 TGAATGAGCAAAATGTATAGTATATTAAACGGTGGTTAAGTGCCATTAGAGAAAGAGAAG 29900051

29900052 GAGGAAACTCAGGGTGGGGGGATGGCAAATGTGATCTTACAGGGGGTTCAGGCAAAGGTG 29900111

29900112 ACATGGAAATGAGACTGGAAGTAGTGAGTGAGGAGCTGCATGGCTGTCCAGAAGCAAGGA 29900171

29900172 GCCACCTGAAAAGGCTCTGGGGCAGGAGTGTGCTGGGCACGTGAACACAGCCAGGGTGGC 29900231

29900232 TGGAAAGAGCTACAGGGGAAAGA

C9-OFF3

>chromosome:GRCh38:12:71064227:71064849:-1

71064849 TTGTATCTAAGTCTGCTGAATCCGTAGCCCCACTTGTCAAGTGACAGAATCTCGGCTCTT 71064790

71064789 TCCTTTCTGGGAGGATTGTCCTACACATCATTCATACCCCTTCCAGCTTCTATCATCCCT 71064730

71064729 CAGCTGCAGCTCTTCCCACTGTCTATCATGTCTGTTGCCTACTATGGATATACTCATTGC 71064670

71064669 CTGTGCTTCCTCCACAGTTCTGCTGATTTATTTTACCATCAAAATAATCAATATTTATTG 71064610

71064609 AACACCTACTATTTGCCACACATTCTTCTATGGCCTTAGGCTGCAGCAGTAAACAAAACA 71064550

71064549 **GACAAAAGTTCCTGCTCTCCTGG**AGCACTGGGGGGAAGCAGACAATAATATAACTGTCAT 71064490

71064489 AAGTAATAAGAAACATAATACATAATACATTGCCTATAGAAAGCATTATGTGCTATGAAA 71064430

71064429 AACATTCAGAAGGGTAATAGGAATCAGGGATGTTGGGGAGGGAGGTTGCAATCTGAAACA 71064370

71064369 CAATGCTTATAGTAATACTCATTGACAAAGCGATACCTGAAATGAGACTTAGAGGAAATG 71064310

71064309 AGCGAGTTATTCATGTGGATACCTGAGGCAGACGAACCGTACAGTGTAAAGACCCTACAG 71064250

71064249 GGACAGGGCGTTGTTTAAGAATT

C9-OFF4

>chromosome:GRCh38:19:33751516:33752138:-1

33752138 GGCGTCCAATACCCGCTAGGCGGAGCCTCCCCTTCGTCCTGGGGCTCAGGCAGCTGTGGC 33752079

33752078 AGCAAATCTCACTCCAACCCCTGGGCAGGAAATCTCATCCAATCCTAGGTGCAGGGTTGG 33752019

33752018 TGGGGTCTGAATGGCTGGCCAAGCAGGGCCCCTCCGCTGCCCAGAAGGGGGAAACACCAC 33751959

33751958 GCTGCTACCCCGATCCCTGACCCCTCCTGGCGCCGTTGGAGGGAAAACACTCATTCTGTC 33751899

33751898 ATTGTTCATTTGGGAGCTGGTGCCGCAGGCCCCATGCTGAGGGGTGTTAAAGGCTGGACA 33751839

33751838 **GAGAAATGATCCCGCTCCCGTGG**GGGCTTCAGCAGGATAGGTTTTACTATCCCTGTTTGG 33751779

33751778 CATGAGGGCACTCACTGAGTCTCAAAGAGGTTAAGTGGGTCACCCTCATCCACACTGCTG 33751719

33751718 CCCAGGTAGGCTGGCAGAGTGGTGGGTCCAGGCCCGAGCTGTCTGATTCCATGTCCTTGC 33751659

33751658 TGTAACCACTCTCTGATGCTGCCTGCCAGTGCCCGGGGACGGTGGGCCGGTCAGGCAGGA 33751599

33751598 GGCTGGGGGGTTCACAGAACGTCTCACTATCATTAAGCCAGTGTCCACCATCAGAAAAGC 33751539

33751538 TGCCAGGGTCTTGGCCTTGGGCT

TALE6-OFF1

>chromosome:GRCh38:1:162224446:162225060:1

162224446 AAACTTAGGACATTATTTGATGTTCAAGGTGGTTGAGCTTTATAGGTGCAGAGGCTTAGA 162224505

162224506 ATTGGAATGCTGGGGGTCAGGAGAAAGGTCACATAGAAAGACAAGTGCCAGGTTGGATGC 162224565

162224566 AGGTAGCTGTGGCACTAAGAGCAATATCTCTTCTGATGGAAAGCTTTACTTTAACTAATT 162224625

162224626 AAACATGAAATAAAAGTGAAAAATTGTACTGAACAAGAAGACAGAGGAAATGCTACAGAC 162224685

162224686 TCATCCCCTGAGCCTGATTTTATCCAGAGTAACTTAGGGCAGGAAAGGAGAGAAGTTAGA 162224745

162224746 **GAGGACAAGTAGTGC**CTGTCTTTGTTCCCAGGAGAATTGACCAGAATCTTCCCTGTTGAT 162224805

162224806 TTTGGCTAAGACTGATGTTGTTTCCAGGATGAGACCCCATGTCCTGAGGGTCACTGCCTT 162224865

162224866 ATCAGAGATGGGCTGGGGGCTCTTGGGTCAGTGAGCAACTCAGCCAGTAAAAGCAGGGCC 162224925

162224926 CCCGGGAAGTCTTAAACAAAGAAAGAAGTGAGCATTGAAAAAGAAGCATCTCAGTAAAAA 162224985

162224986 TAAGGTTCTAATGAGCTTTCTTCTTGGCAACCATTCTCTTCCCAGTGACATGTCTGAGTC 162225045

162225046 TCAGAAGTCCTGCAG

TALE6-OFF-2

>chromosome:GRCh38:5:148295355:148295969:1

148295355 AAACCAATACAAACTCATTTCATCTTTATTTCTTCTGAGTCAATTTTATCATCATTTGTT 148295414

148295415 TTACTACTTGTACTGTACTCAGAGTAGGTGTGTCTATTTCTTGTTAAAAAAAATCCAGTT 148295474

148295475 AGGCTTATTTTTCTATATATAAATTATGGGTAAAATGAATACAAATTTGAGTTTCCTAAT 148295534

148295535 TTATCTAATAAGGTTAAATAAATAAATTTTTAAAACAGGTTTATCTAAAATTAAGATAAT 148295594

148295595 TCTAAAAATTAAAAAGATATAATATAAATTATTCTATTTTTTTGGACTGTTATACATTTT 148295654

148295655 **GAGGACAAGTAGTGC**TGGAAGAAGGATGAGCAAAAGCCCTAATTTGTACCATTTGTTCAT 148295714

148295715 TTTCGCAGGGTAAATACTCTCAAGTGGTCAAATTCAAGCTGCCCTTAGCAATCAGTTTGC 148295774

148295775 AAAATCCCTGAATACTTACTTGAAAACCAATTCTCAAGGGCTGATGTAAGGTGACTCCAG 148295834

148295835 CACACTACCTTAAGTAAAAAAACTAAGCTTAAAATACTACATATTGTATCTTATCGAAAT 148295894

148295895 TTAAAGCTATTTAATTCTTTCGCTTTGTTAAATAGCCTATTAAAAAGATGGAAAGACAAA 148295954

148295955 CTCTAGACTGCAAGA

TALE6-OFF-3

>chromosome:GRCh38:8:113645255:113645869:1

113645255 TCCAATGTGGGAAAGGAAATATCACAATAGGGAGAATACTCCAGATCAAAATATCTGCAA 113645314

113645315 GGATTAGGCAAAAAAGATTTCTGTACTATAGGGAGGAGTGAACAAAGAAAAAAATCAGAT 113645374

113645375 GTCTTGCTGTACTAGCAGATCAGAGAATGATCATGAAGCCAGCCTATTCTTGGGAGGGAC 113645434

113645435 TGCTAAGGAGGGGATGTATTTTGTCTCTAGCAGGGTGAAGACCAAAATGCAAGGACCTAG 113645494

113645495 GGGAAAAGAAGATACTTAACCAAAGTTTCATTGACAAGTATTTTGTTCTGATTAAACAAT 113645554

113645555 **GAGGACAAGTAGTGC**AGCTAATCATATATGAGGCAAAGAATGGCAATTTGGAGAGTCTGC 113645614

113645615 TTTTGGCCTTGTCACAGGTGAAAAAAAAGACAGCATCCATGAATCTTCTCTAAGGCATAT 113645674

113645675 AAGAAGGGTTGTTCTTTGCAGTAAGTCCTTTTAGGGAACACAAAATGTTAGAGGAATTTC 113645734

113645735 TTAATCTTTGCTGTTTTCCAGGATCACAAGTGACCAAGACAGTCATAATATTCTCCTCAG 113645794

113645795 CTTGACTCAACTTTAGCAAGCTTCTTATGACTCTGGGCAGCTGACCTTCCTCTTCTTAGA 113645854

113645855 AAATTTCTTCAGAAA

TALE11-OFF- 1

>chromosome:GRCh38:15:21003144:21003758:1

21003144 CCCTCCACTGAGAGCTCAGCTCCTAGGCTGAGGAATAAAACAGCTCAACTTTGTCTACAC 21003203

21003204 CTGCAGAACCTTGTTTAGGAGCTCTGTCCCAGGAGAGAGGGGGCAATGGAATTCAGTCAT 21003263

21003264 AAAATATGATCCTTAATTAGTCCTAAAAATCCTAACTTCAGTAACAGCAGATTGTGGACA 21003323

21003324 AATTGAAAGCCTGCCAGTGCTCTCAAAAACAGTGGATGGTGTGGTGGAAAGCCCTTGGAA 21003383

21003384 GGAGACGGGTGGATGCATGGGAGATGCAGGCTACACTGCAGGGCTGCTGGCTTGCAGGAG 21003443

21003444 **AGAACCGAGGACAAG**GGAGAGCTGGGGAAAGTTCTCTTGTGGTTGAAATAAATGCCAGAC 21003503

21003504 ACTCTTCAATGGAGCCCATGTTTGTTTGGTTCAGTCTGTGAAGTAATTCAAACCTCAGTG 21003563

21003564 CATGATTGAAAATAGTACAATTTTCCATCTGCAAGTGGCAGAGCTCAACATCTGGGTCTG 21003623

21003624 GTCAGGAGAGAGACAGAGAGAGCCCAGCCCAAACCACTGACACCTGGGGTTGACAGTGCT 21003683

21003684 GCTTACAGATGTGTTCCTTTGATCTTTGAGACTGGTTTCTCTCACTTAGCATAATGCCTG 21003743

21003744 GAGTTCAACTGTAAT

TALE11-OFF-2

>chromosome:GRCh38:16:74764752:74765366:1

74764752 TTAGAGAAGATTGAGAACCACTGTTCTGAGTCTATGTGCTTTAGAGAATTTTGCATGAAA 74764811

74764812 AGACACCAGAAAGAGTAAACAGGACTAGAAACCCAGGCAACCTGGGACATACCTATGAGT 74764871

74764872 GTCACCCAGGTACTTATAGCTACATGAGGACTGGGGGGGTCTTAACCCTGAGAGCAATGT 74764931

74764932 GGGTCTTGATTGAGATTTTCTATGGTTAACTTCACTTCTAACCCCTGAAGGAGACTCATG 74764991

74764992 GGACGGGTAGACTGTAAGAGCCTGCTAAGGGAGAGGTTTTGCTAACTCTCTCCGGCTGCA 74765051

74765052 **AAGAACCGAGGACAA**AGGCAGAAATGAGTGACTTGCTTGATGGTTCTCCATCCTTTCCAT 74765111

74765112 CAACTCCCAACCTTTTAACAAGACACAGAAAAGAGATTTTTCTTAATCTTTTTTTTTTTT 74765171

74765172 TTGATACAAAGTCTCGCTCTTGTCCCCCAGGCTGGAGTGCAATGGCGCGATCTTGGCTCA 74765231

74765232 CTGCAACCTCCGCCTCCTGGGTTCAAGCGACTCTCCTGCCTCAGCCTCCCAAGTAGCTGG 74765291

74765292 GATTATAGGCATGCGCCACCACACCTGGCTAATTTTTGTATTTTTAGTAGAGACGAGGTT 74765351

74765352 TCACCATGTTGGCCA

TALE11-OFF-3

>chromosome:GRCh38:1:76872151:76872767:-1

76872767 ATGATTAGCATTCATTTCAGTCATTCATTCACAACTCAAATGTCATAAAATGTGGTCAAA 76872708

76872707 TAAATTATGTAAACTTGTGTTTGATAATTTATATACTTTTCTAAAAGGGGAATACTCATC 76872648

76872647 CCATAATTTTAAAATAAGTTAGATGGCATTTATCCAAATTCCGGTTTTGGATGACTATTT 76872588

76872587 TTCAGTTCTTGCATTCACTTGTCTTACCACTCCAACCAGATAGTTACCAGTTAATAGTAA 76872528

76872527 TAGTACTATTACCACTAAGCAATTTTGATTTGTCTGAATAACTGAGTGCCTAAAAATGGA 76872468

76872467 **AAGAACCAAGGACAAGT**ATGTTATTGGTATGTGTAAAATCCATCCCTGGGGGCTACTTCT 76872408

76872407 GAAGCAAAGAGATAGCTGCTTAGCTGTAGACCCCATTGATCACCGCCCTTTCATAAGCCA 76872348

76872347 TTTCAGTAAAGGGCAGTCTGGGTGATAGTGGGCCTTCTGTGATAAGAGCCTGTCCTCTAG 76872288

76872287 AAATGGGAATTAGCTCACGGTTTCATTTCACAGGGTCTACCAGATGGCAAATGACCTCAT 76872228

76872227 CTACCCAGACGAGATCCAGACTGGAAACTGAACTGTGTTCCATGTTCCCTTCCATTCTGC 76872168

76872167 TATAAGACTTGGAGCTT

1. HEK2

| TaC9 target locus | On target sites | gRNA off target sites | Tale off target sites |
| --- | --- | --- | --- |
| HEK2 | 1 | 3 | 3 |

On target : **GTTCTTGTTCTCCTATA**CCAGGA**GTAGAAAAAGTATAGACTGCAGG**

>chromosome:GRCh38:14:88099561:88100183:-1

88100183 GTAACTATATGCTCTCTGATTCTCCTATTAGCAATGATGAGAAAGAAAGTTTTATGGCCA 88100124

88100123 GTATCTAGAAATCATGTATAGTGACTCTCTGCCTCTGCCTCACATGAGTTCACATAAAAC 88100064

88100063 ATATACAAAAAAGTTCATCAGAAAAATACAAACTCATTTTTATTTACTTGGGTTTTGTCT 88100004

88100003 TAAGCCTTTATGAGGAACCATCTACCCATGTGCCTGACATAGGTCTTATTTTTCTCCTTA 88099944

88099943 GTACAAAGGCAATTAAAACAATCTGAGTGACTTCTTT**GTTCTTGTTCTCCTATA**CCAGGA 88099884

88099883 **GTAGAAAAAGTATAGACTGCAGG**CAAATGCTTTCCATCACCTGTTTTTTTACTACTATTC 88099824

88099823 TATTGAATTATAATTCATATACCACATAATACAATCATTTTAAGTATATAATTTAATGGT 88099764

88099763 CTTTAGTATATTCATAACGCTGTGTAACCATAATCACCATAATTAATTTTAGAACATTTT 88099704

88099703 CATCACCCCCCAAAGAAACCCTGCACCCCTGAGTTATCACCCTCCAATCCCCACTTTTTT 88099644

88099643 CTGCACCCCACGCTAGGCAATCACGAATTTGCTTTCTGTCTCTATAGATTTGCCTTTTCT 88099584

88099583 GAACATTTCATATAAATGGAATC

C9-OFF1

>chromosome:GRCh38:8:47998021:47998643:1

47998021 ACTACAGAGAGTAAAGAAAGCCAGAACATAAGGAATCTCAGACCACTTCCCATTGCGGTG 47998080

47998081 ACAAAAGTTGTCTTAAGTCTCTGAGCACGTTGGAATCGAAAGTGCCATTTTCGGACCATT 47998140

47998141 GGGAGTCATGGTCTAATTTGTATTGAGGTCACGCGGTATTACAGTAAAAGATAAGCCTTT 47998200

47998201 TAGGGCAGATTTCTGAACGGAGGCCAAGAGTATTGAGATTGCATAGGAGGCACCCAAGGC 47998260

47998261 AGGTAGTTTTAGAAGGAGCAGACTGAAAGGCTCCTATTATGAATGGAAGTAGGGGCGCAC 47998320

47998321 **GTAGAAGAAGAAGAGACTGCCGG**TGACAAGGACGTGGAAGAGGGCGTTCCCTTTCCTGGG 47998380

47998381 GAACTTGTCTGGAATAGAGAAGGTAACCGCCGTGTCAGGTGTCCCTGACACAGAGGAACC 47998440

47998441 AGAAGCCTGGAGGCCGGAGGAAGCCCTTGACCCAGGGCTGGGTCTTTTGGAAACTGAGAG 47998500

47998501 ACAGGCGGTCAAGGGTTTCGGAGAACAGCAACAGTCTCTTTTTACTCACCCTGGCGGAGG 47998560

47998561 CTTTGATGGTGGATGAAGTCGTCAGCAGTGGGAGAGTCTAGGAGATTCTCTGGGACTTCG 47998620

47998621 GCAGGTTCAGGGAAGGGGAGAGA

C9-OFF2

>chromosome:GRCh38:17:70657080:70657702:1

70657080 TACCGTCTAACTTTCATCTATATTGATGTTTTTTTCAACAAGAGCAAACTGTTTTGGAAA 70657139

70657140 AACTGTCTCAAATATCTTTACCACGTTGGTCTATGATCACAGGGTTTACAATTTCCATTC 70657199

70657200 TTTAACAATACTTTTTCTGAAATGGCTCTTTTAGAAAAAGACGAGTTAAACTATTTTACT 70657259

70657260 GTTGTATGTATATAAAATCTGAGAATATTGGTAATTTACGATGGGTCTGGTGATGCTAAA 70657319

70657320 ACTACATACATATGAAGATTAAAGCAGCTCTTTAACATAAAGTTAGGTGGATATGACTTG 70657379

70657380 **TTTGAAAAAGTATAGACAGCGGG**CACAGCTTTTCACAGACTACAAAGATGATATTGCTGA 70657439

70657440 TATGTAAGATGAAATGGCAATATGACAAATACAGGCAGGCTGGGCGCAGTGGCTCATGCC 70657499

70657500 TGCAATCCCAGCACTTTTGGAGGCTGAGGCAGGTGGATCACCTGAGTTCAGGAGTTCGAG 70657559

70657560 ACCAGCCTGGCCTGCCTGGCCAACATGGTGAAACCCCATATCTACTAAATATACAAAAAA 70657619

70657620 TTAGCCGGGCGTGGTGGCAGGTGCCTGTAATCCCAGCTACTTGGGAGGTTGAGGCAGGAG 70657679

70657680 AATCGCTTGAACCCGGGAGGTGG

C9-OFF3

>chromosome:GRCh38:3:132054020:132054642:-1

132054642 AGCCAACCAATCCTTGATTGTTTAAGCTAGTCTGCATTCATTGTTCCTTATAACCAAAAA 132054583

132054582 TATTCCAACTTGCTTTCTTGTATACTAGTACTAAGAATTATGAAAATTTGTTTTGGGGTT 132054523

132054522 AAAGCATGCAAGAAGAATAACAACATATTATTTATTAATTCAACAAATATTTATTGAGAG 132054463

132054462 TCTACTGTGTACAAAGCTCCATTCTAGCCTCGGGTATAGAATGGCAGATGAAATAGGTAG 132054403

132054402 TAAGTTAAGGAATACAGACAATTAAGCCAGCAAATACAATACAAGGTGGTAAATGCTATA 132054343

132054342 **GTAGCATAAGTATAGACTACTGG**TAGGCATATAACAAGAGCATATTATTAAGTGGGTCCC 132054283

132054282 TGACCCCCGAGTAGCCTAACTGGGAGGCACCCCCCAGTAGGGGCAAACTGACACCTCACA 132054223

132054222 TGGCCGGGTACCCCTCTGAGACAAAACTTCCAGAGGAACGGTCAGGCAGCAACATTTGCT 132054163

132054162 GTTCACCAATATTCGCTGTTCTGCAGCCTCCGCTGCTGACACCCAGGAAAACAGGGTCTG 132054103

132054102 GAGTGGACCTCCGGCAAACTCCAACAGACCTGCAGTTGAGGGTCCTGACTGATAGAAGGA 132054043

132054042 AAACTAACAAACAGAAAGGACAT

TALE-OFF1

>chromosome:GRCh38:14:42634709:42635325:-1

42635325 AAAGCTCACACATAACATAGATTTTTTCTCTTCCCAGTATGGACCCATTCTTAAATGATT 42635266

42635265 TGAGCTCCAAACAGGCAATAGAGAAGACTAACAAACCAGATTACCTGTTGTCTCTCACTC 42635206

42635205 AATAAAGTGAAGATCTCTATAAACTATTCATCTGTTTATTGAGATCACCAAATTGTTAGA 42635146

42635145 CTTTAAACCAAATTCCCAATATAGGATAACTTATTAGCTATTAAAAATCAAAAATAAAAC 42635086

42635085 ACAAAGTACAAGAAACAAGTACCAAAAATCAAGCCAGTAGTCAAAAATAATTACAATCAA 42635026

42635025 **GTTCTTGTTCTCCTATA**GATGAACAAGACTCCTCTTTGGATCCTGGGTCACCTGTCCAGA 42634966

42634965 GATGATCAAACAGGCCTGGGTTGAGTGAAGGGGGATTTGAAAATGAGGTTCTGATTGTGA 42634906

42634905 GCCATGTTATAAAATATGTGAAAGAAAAAATGCATTAAATAATTCAGAAGAAATTTTTTT 42634846

42634845 TAGCCTATTGCAATAGTAAGAAAGTTCATTTCTGAAGAAAATTTTCAGAGTAAAGGAAGG 42634786

42634785 CAGTCTGAGGTTCTACAGAGGCAGTCAAACAACGGAGTTATGAGGAAGATTCTTAGCCAT 42634726

42634725 GAGGAATGGTGATCCCT

TALE-OFF2

>chromosome:GRCh38:4:93878308:93878923:-1

93878923 AGGAAAAGTTGCCACAGAGAATGAAGAAAATCAAGGAAAAGGTGTTGGTGAAATGAAAAC 93878864

93878863 CTACAAAGAAAGAGTTGCAACAGCAGTCAGGAGGTCAGTGTTTCAGGATTAAACAGTGTC 93878804

93878803 CATTAGCTCTGTCACTTAGGAGGTTAACTTGTCCTTACAGTGAACTTTCAGTGCAGTGAT 93878744

93878743 AGGGTGAAAGTCAAATTACACTGAGTTGAGAAGTGAGTTAGAGGTGGAAGAAAGTTAAAG 93878684

93878683 AGTAGAGACCATTTAAAAGAAATTTAGATCTGGACAAAAATTGAAAACCAGGGTAGTATA 93878624

93878623 **TATAGGAGAACAAGAA**TGTGGAAAGCTTTTTTGTCTGTTTGTTCAGTTTTTTAGATTTGT 93878564

93878563 AATTTGAGGGAAGAGGGGATAATGGACGATCCCTGAAAAATTGAGATGTGATTGTGAAAA 93878504

93878503 CAAGTAGATTCTTAAATTAACTTATTTTTATTTTCCTAACAAAATTCAATTTTCTTCTCT 93878444

93878443 TAAAGCTTCTTTTTGTTTGTTTGTTTGTTTGTTTTGTTTTGAGACAGGGTCTTGCTCTAT 93878384

93878383 CGCCCAGTCTGGAGTGCAGTGGCGCGATCTCGGCTCACTGCAAGCTCCGCCTCCTGGGTT 93878324

93878323 CACACCATTCTCCTGC

TALE-OFF3

>chromosome:GRCh38:6:98342174:98342790:1

98342174 GTGGTAATCATTTCACAATATATACATGTATCAAAGCATTACATGGTACACCATAAAAGA 98342233

98342234 CAATTTTTATTTGTCAATTATACATCAATAAGAAATGTTTTAAATTAATACAAATAAAAA 98342293

98342294 CATTTAACTTCTCTATGGGCAGATACAGAAGAGCCTTCATTGGTTATTAGTAGATTGTGT 98342353

98342354 TGCTTCTTCTAAGCCAAATCTTTCTTAATTATGTAGTAGTGGCATGGCTGACTTCCACTT 98342413

98342414 CCACTTGTGTCTTCTAGAGAGCCAATCATAGCATTAGTGTCAGACACAGCATGCAGGTGG 98342473

98342474 **AATAGGAGAACAAGAAC**ACAGGTTTCATGCTTACAAATGCACCTTCTCAAAGAAGAGGTG 98342533

98342534 TGATAGGTAACCTTGAACAAACTTCTGACTTCAATTCTGAGCCACAATCCTATGACCTTG 98342593

98342594 ACCTTCTTCTCCTTCTCTTTACCAAGGAGGGACCATAATAAGGTATTCACTCTCCTAAGA 98342653

98342654 CATTAGCAGGTGTTTAAAGGATCAATGTTTCAAAAGCATCCTGGGGAGAAAAAAATGACT 98342713

98342714 TGGAAACATGTAATAGTTTTATTAATTCACCTTCTTTTTAATGAATCATTAAAACTGAGC 98342773

98342774 TTAGAGCAGCCTACCTG

**The loci information of Figure S5 and S6**

1. HEK3

On target : **atacatgtttaagga**atttgaggaaaaaaaggaagaaaattagg

>chromosome:GRCh38:4:131198835-131199435:-1

131199435 CATGTGATACATAATGAAACCACAATATCACCTGTCAAATAAATACTAAAATAAAAAATA 131199376

131199375 AAAATAAACCCATTTACTTTCACCTTAGCATCCACGCTATTAACCAATACAACAATTTGC 131199316

131199315 CTATAACCCAAATTCCACAGAAAGACCAGAAATAGAGAAAACCTAAAAAAAAAAAATGAT 131199256

131199255 TGAAAGAGTGAATTTTCAAACCTTAAGAGAAAGATATATATAGAGAGAATCAAATACATG 131199196

131199195 CTTCTAAGGATAGGAAGTAAAAGCAGAAGGTAT**ATACATGTTTAAGGA**ATTTGA**GGAAAA** 131199136

131199135 **AAAGGAAGAAAATTAGG**TCACAACCACAAGTAAAACATGAATTAAAGCTATAATGTAGTA 131199076

131199075 GGATAATTTTTCTACTTTCCTTATTACAAATCCATTATTTTTGATTAATGACTTTTTAAG 131199016

131199015 AATGTCTCTTTACTTTTGGCCACAGATTGAGGTCAGTGGTATGAGGAATAACAAGCAAAA 131198956

131198955 CACAGAATTATGTTTACAAGTAGAAATCTAGTAACTGTAGACTTGTAAAGCACATATTTT 131198896

131198895 ACATTGTGTCACAGAGTTCTGTTTCAATTTTGTTAATTATATAAGGAAAATAAAATAGGTT

1. HEK4

On target : **tttatcccattgcttta**aagaaagaaagaaaagtaagaaaattggg

>chromosome:GRCh38:5:154082128-154082739:1

154082128 AGCTACCTTCTTTTCTATGTCCACAGACTATCTTCACCTGAATAGACACCCCCTCCGGGC 154082187

154082188 ATCTCCTGAACCAGCCTCCCTGTGGAAGTTGTGTTATGGACATATATATGGTAAATGAGA 154082247

154082248 GTGTCACTGTTCAAAGGTATCACCAGTCTTCTAGGCAACAATAAATTCAACCTGGTGGTG 154082307

154082308 TCTCTGTCCATCTTATATATACAGCCTTTTGGATAAGAACATTTCACTACACATAATGCT 154082367

154082368 GAC**TTTATCCCATTGCTTTA**AAGAAA**GAAAGAAAAGTAAGAAAATTGGG**TAAAAGTTCTT 154082427

154082428 CTCATCCATCCACTTATTAACTAGGTAACCTTTGATAACTGACCTAACCTTTATTTTACT 154082487

154082488 TAAAAAGGGAAAAGGAAAAGGCCTTCTCTCTGATTTAAAGTCATGTGTGTCCTTCATAGA 154082547

154082548 AAAATTTAGAAAACTTAGAAAAGTACAATAATCCAGACACCCTCACCTCAACCAGTACTT 154082607

154082608 TTTGGAAAATTTCTATCCAATCTTCCTTTTCTCTATGCAGATATTCTTAATTGTGATAGA 154082667

154082668 CATTAATTGTGTTCATCATCTGGTCCAGATAATTATCTGGTATGAAGAATGGCCTACCAG 154082727 154082728 ACCTTAAATCTG

1. Nanog

#1 On target : **ggaagctgctggggaag**gccttaatgtaatacagcagaccactagg

#2 On target : **gaagatgagtgaaactg**atattactcaatttcagtctggacactgg

>chromosome:GRCh38:12:7794751-7795356:1

7794751 AACCTTCCAATGTGGAGCAACCAGACCTGGAACAATTCAACCTGGAGCAACCAGACCCAG 7794810

7794811 AACATCCAGTCCTGGAGCAACCACTCCTGGAACACTCAGACCTGGTGCACCCAATCCTGG 7794870

7794871 AACAATCAGGCCTGGAACAGTCCCTTCTATAACTGTGGAGAGGAATCTCTGCAGTCCTGC 7794930

7794931 ATGCAGTTCCAGCCAAATTCTCCTGCCAGTGACTTGGAGGCTGCCTT**GGAAGCTGCTGGG** 7794990

7794991 **GAAG**GCCTTA**ATGTAATACAGCAGACCACTAGG**TATTTTAGTACTCCACAAACCATGGAT 7795050

7795051 TTATTCCTAAACTACTCCATGAACATGCAACCTGAAGACGTGT**GAAGATGAGTGAAACTG** 7795110

7795111 ATATTA**CTCAATTTCAGTCTGGACACTGG**CTGAATCCTTCCTCTCCCCTCCTCCCATCCC 7795170

7795171 TCATAGGATTTTTCTTGTTTGGAAACCACGTGTTCTGGTTTCCATGATGCCCATCCAGTC 7795230

7795231 AATCTCATGGAGGGTGGAGTATGGTTGGAGCCTAATCAGCGAGGTTTCTTTTTTTTTTTT 7795290

7795291 TTTCCTATTGGATCTTCCTGGAGAAAATACTTTTTTTTTTTTTTTTTTTGAAACGGAGTC 7795350

7795351 TTGCTC
